# SCD Inhibition Protects from α-Synuclein-Induced Neurotoxicity But Is Toxic to Early Neuron Cultures

**DOI:** 10.1523/ENEURO.0166-21.2021

**Published:** 2021-08-07

**Authors:** Justin W. Nicholatos, Joost Groot, Shekhar Dhokai, David Tran, Lori Hrdlicka, Thomas M. Carlile, Melissa Bennion, Isin Dalkilic-Liddle, Warren D. Hirst, Andreas Weihofen

**Affiliations:** 1 Neurodegeneration Research Unit, Biogen, Cambridge, MA 02142; 2 Biogen Postdoctoral Scientist Program, Biogen, Cambridge, MA 02142; 3 Translational Genome Sciences, Biogen, Cambridge, MA 02142; 4 Biologics Drug Discovery, Biogen, Cambridge, MA 02142; 5 Bioassays and High-Throughput Screens, Biogen, Cambridge, MA 02142

**Keywords:** cell-based assays, lipids, neurotoxicity, Parkinson’s disease, SCD, synuclein

## Abstract

Here, we report the independent discovery and validation of stearoyl-CoA desaturase (SCD) as a modulator of α-synuclein (αSyn)-induced pathology and toxicity in cell-based Parkinson’s disease (PD) models. We identified SCD as top altered gene from transcriptional profiling in primary neurons exogenously expressing αSyn with the amplified familial PD mutation 3K. Thus, we sought to further explore SCD as a therapeutic target in neurodegeneration. We report that SCD inhibitors are toxic to early human and rat neuron cultures while displaying minimal toxicity to late cultures. The fatty acid product of SCD, oleic acid (OLA), fully rescues this toxicity in early cultures, suggesting on-target toxicity. Furthermore, SCD inhibition rescues αSyn 3K-induced toxicity in late primary neurons. We also confirm that SCD inhibitors reduce formation of αSyn accumulations, while OLA increases these accumulations in an αSyn 3K neuroblastoma model. However, we identify a caveat with this model where αSyn 3K levels can be suppressed by high SCD inhibitor concentrations, obscuring true effect size. Further, we show that both SCD1 or SCD5 knock-down reduce αSyn 3K accumulations and toxicity, making both a putative drug target. Overall, we confirm key findings of published data on SCD inhibition and its benefits in αSyn accumulation and stress models. The differential neurotoxicity induced by SCD inhibition based on neuron culture age must be accounted for when researching SCD in neuron models and has potential clinical implications. Lastly, our gene profiling studies also revealed novel putative genes connected to αSyn neurotoxicity that are worth further study.

## Significance Statement

There is no disease-modifying therapeutic for those suffering from Parkinson’s disease (PD). Recent research has shown stearoyl-CoA desaturase (SCD) inhibition to ameliorate α-synuclein (αSyn)-related pathology and neurotoxicity in preclinical PD models. The use of neuronal cell models to study PD-related pathology is critical for developing putative therapeutics. In this work, we demonstrate important caveats in cellular PD models when studying SCD inhibition. We also independently identified SCD and other genes as potential targets for PD. Overall, this work supports SCD as a clinical target and adds important considerations for studying SCD in *in vitro* models.

## Introduction

As the population becomes increasingly aged it is ever more important to develop therapeutics for age-associated neurodegenerative diseases such as Parkinson’s disease (PD). PD is pathologically characterized by neuronal inclusions, called Lewy bodies (LBs) and Lewy neurites (LNs), and neuronal loss, preferentially of dopaminergic neurons in the substantia nigra pars compacta. LB/LNs are mainly composed of aggregated α-synuclein (αSyn) and lipid membranes (Fares et al., 2021). Reduction of these hallmark structures are considered a therapeutic approach for a disease modifying therapy for PD and other synucleinopathies. Recent studies have shown that inhibition of stearoyl-CoA desaturase (SCD) ameliorates αSyn-related pathology and neurotoxicity in both *in vitro* and *in vivo* models (Vincent et al., 2018; Fanning et al., 2019; Imberdis et al., 2019; Maulik et al., 2019; Terry-Kantor et al., 2020; Nuber et al., 2021). SCD is the rate limiting enzyme in the production of monounsaturated fatty acids (MUFAs), such as oleic acid (OLA). αSyn can directly bind OLA (Sharon et al., 2001) and the presence of OLA in membranes can enhance their interaction with αSyn (Kubo et al., 2005). αSyn’s membrane binding properties are believed to play a functional role in synaptic vesicle trafficking and neurotransmission (Runwal and Edwards, 2021). These αSyn-membrane interactions can alter αSyn aggregation kinetics based on lipid membrane composition (Galvagnion et al., 2016). Recent studies have implicated OLA in promoting αSyn-lipid accumulation pathology (Fanning et al., 2019; Imberdis et al., 2019). Altering cellular lipid profiles, and more specifically lowering MUFAs such as OLA through SCD inhibition, may thus be a viable therapeutic approach to target αSyn pathology in PD. Yumanity Therapeutics is testing a SCD inhibitor in clinical trials for PD and has another SCD inhibitor in their pipeline for LB dementia (Yumanity Therapeutics, 2021). Scientists from Yumanity initially discovered SCD inhibition as protective from αSyn-induced toxicity in yeast (Vincent et al., 2018).

Many of the studies demonstrating benefits of SCD inhibition in PD used an “amplified” familial αSyn E46K model, which harbors E35K + E46K + E61K mutations (αSyn 3K). This model is advantageous in its ability to recapitulate features of human PD pathology (Ericsson et al., 2021). Transgenic mice with αSyn 3K display dopaminergic neurodegeneration and develop PD like motor symptoms that are responsive to L-Dopa treatment (Nuber et al., 2018). LBs are not only loaded with αSyn but are full of membranous lipids and vesicular structures (Shahmoradian et al., 2019). Transgenic mice and cellular models that express αSyn 3K recapitulate this lipid rich vesicular clustering during accumulation formation and exhibit cytotoxicity (Dettmer et al., 2017; Nuber et al., 2018; Terry-Kantor et al., 2020). E35K, E46K, and E61K αSyn mutations alone and in combination have been shown to have altered lipid binding properties (Rovere et al., 2019), which likely contributes to their pathogenicity.

Here, we report the independent discovery of SCD as a top hit identified in a transcriptional profiling study investigating αSyn neurotoxicity. This internal discovery and the active clinical interest spurred us to validate key published findings and expand data on SCD inhibition in PD models. In addition to supporting much of the published data on SCD inhibition in cell-based models, we also identified important caveats that must be considered when studying this target in neurons and αSyn overexpressing systems.

## Materials and Methods

### Antibodies

Antibodies used were MJFR1 (Abcam, 138501) or syn-1/Clone 42 (Biosciences, 610787) for total αSyn (Abcam, 138501), EP1536Y for pSer129 αSyn (Abcam, ab51253), MJFR14-6-4-2 (Abcam, 209538) for oligomeric/fibrillar αSyn, C4 for loading control actin (Abcam, ab14128), and CD.E10 for SCD (ThermoFisher Scientific, MA5-27542).

### SCD inhibitors and OLA

MF-438 (MF; EMD Millipore, 569406) and CAY10566 (CAY; Ambeed, A698046) were resuspended in DMSO and dispensed to cell cultures via a HP D300e digital dispenser. OLA BSA conjugate (O3008-5ML) and fatty acid-free BSA control (A8806) were purchased from Sigma. OLA and BSA control were diluted in culture media before addition to cell culture (50% exchange).

### AAV generation and transduction

cDNAs for human αSyn, αSyn 3K (αSyn E35K + E46K + E61K) and αSyn 3K S129A were synthesized (Geneart, ThermoFisher Scientific) and subcloned into AAV entry vectors under the CAG promoter. Empty vector without insert control (EV) vectors were packaged into adeno-associated viruses (AAVs) using AAV9 capsid serotype (Packgene Biotech). Control AAV (EV) was generated using entry vector without cDNA insert. Purity was determined using SDS-PAGE followed by Coomassie staining. AAV genome copies were quantified by determining copy numbers of AAV2 ITRs via qRT-PCR. Cells were transduced at a multiplicity of infection (MOI) of 80K for primary cortical neuron cultures and 250K for iPSC neurons, unless stated otherwise.

### Primary neuron cultures

Mouse cortical neuron cultures were isolated and homogenized from timed-pregnant C57BL/6J mouse embryos on embryonic day (E)17 according to Institutional Animal Care and Use Committee (IACUC) guidelines. Rat cortical neurons cultures were generated from timed pregnant Sprague Dawley female rats at gestational day E16. Briefly, cortex tissues were dissected on ice in HBSS without calcium or magnesium (Invitrogen, catalog #21-022-CV), carefully separated from the meninges, washed 3× in ice-cold HBSS, and then incubated at 37°C with 0.25% Trypsin-EDTA and 1 mg/ml DNase I (Sigma, catalog #DN25-10MG) for 15 min. Cell were resuspended in Neurobasal media (Invitrogen, catalog #21103) containing 10% fetal bovine serum (FBS) and 1× GlutaMAX (Invitrogen, catalog #35050) and filtered through a 100-μm cell strainer. Neurons were plated at a density of 16,000 (imaging) or 50,000 (CellTiter-Glo) cells per well in Corning Biocoat Poly-D-Lysine 96-well plate Cellware (ThermoFisher Scientific, catalog #356640) with black wells and clear bottom with lid; 1.5 h later, plating media were aspirated and replaced with neurobasal feeding media supplemented with 2% B27 supplement (Invitrogen, catalog #17504-044), 1× GlutaMAX, and 1× pen/strep (Invitrogen, catalog #15140122).

SCD1 inhibitors, OLA, and AAVs were given on day *in vitro* (DIV)7 for early cultures and DIV18 for late cultures. OLA and AAVs were added with fresh media. Cultures were fixed (4% PFA in PBS for 15 min) for imaging or lysed for analysis 12 d after treatments.

### RNA sequencing (RNA-Seq)

Rat cortical neuron cultures were treated with AAV9 EV or AAV9-αSyn 3K on DIV5. RNA was isolated 6, 12, and 19 d after AAV treatment with a RNeasy plus kit with DNase digestion. RNA quality was measured using the RNA 6000 Pico kit (Agilent, 5067-1513) on a Bioanalyzer 2100, all RNA integrity scores were ≥9. There were five replicates per treatment and for every time point (30 samples total). cDNA was prepared with the SMART-Seq v4 Ultra Low Input RNA kit (Takara, 634891) according to manufacturer’s instructions. A total of 8 ng of input RNA with 1.6 μl of added 1:10,000 ERCC RNA (ThermoFisher, 4456740) was used for cDNA generation, followed by cDNA amplification with seven cycles of PCR using CB PCR Buffer (Takara, 638526). Amplified cDNA was quantified using the High Sensitivity DNA kit (Agilent, 5067-4626) on a Bioanalyzer 2100. Libraries were prepared from amplified cDNA using the Nextera XT DNA Library Prep kit (Illumina, FC-131-1096) according to manufacturer’s instructions; 150 pg of cDNA was used as input, 12 cycles of PCR were used to amplify tagmented cDNA with Nextera Index kit Set A (Illumina, FC-131-2001), and library cleanup was performed as recommended for 300- to 500-bp libraries. Libraries were quantified and QCed on the Lab Chip GX DNA HS Chip (PerkinElmer, CLS760672). Libraries were normalized to 10 nm, pooled, and sequenced on a HiSeq 2500 in paired end mode 2 × 50 bp to an average depth of 8.8 million reads per sample.

RNA-Seq read data were analyzed using a pipeline that used STAR (version 2.5.2b; Dobin et al., 2013) for read alignment and RSEM (version 1.2.31; Li and Dewey, 2011) for transcript read count quantification on the genome (Rattus norvegicus.Rnor_6.0.89) with αSyn 3K vector added. All samples passed quality checks on raw reads with FASTQC (Wingett and Andrews, 2018) and read alignment quality (>90% uniquely mapped paired reads). Normalization and differential expression analysis were conducted with the Bioconductor package DESeq 2 (Love et al., 2014) using a statistical model formulated with time and treatment factors. Sample expression variation was quantified using PCA on gene TPM data which showed sample clustering per factor (time, treatment, no outliers). Thresholds of false discovery rate (FDR) < 0.05 were consistently applied to identify differentially expressed genes. To identify differential expression changes consistent across αSyn 3K treatments and across time points, a rank-based meta-analysis was performed with Rankprod (Del Carratore et al., 2017). Rat genes were mapped to the mouse genome according to the orthologous gene mapping from Ensembl biomart for analysis. Pathway enrichment analysis was performed using Fisher exact tests using the Reactome database. RNA-Seq data were deposited at the Gene Expression Omnibus accession #GSE172385

### Neuroblastoma M17 αSyn 3K-GFP model

Human full-length αSyn 3K with C-terminal GFP (αSyn 3K-GFP) was cloned into pLVX-TetOne-Puro lentiviral vector (Clontech 631849). αSyn 3K-GFP lentivirus was produced in 293T cells by co-transfecting the lentiviral plasmid and lentiviral packaging mix (Virapower, Thermo Scientific) using Lipofectamine 2000 (Invitrogen 11668-500). Stable inducible BE(2)-M17 cells were generated by three consecutive rounds of lentiviral spinfection and selection in complete growth medium containing 1 μg/ml puromycin (Invitrogen A1113803). After third round of transduction, cells were selected in complete growth medium containing 10 μg/ml puromycin and single-cell clones were generated by limiting dilution. αSyn 3K-GFP expression and accumulation formation was induced by addition of 2 μg/ml doxycycline to the culture medium. Multiple single-cell clones were assessed by counting the GFP positive cells and then quantifying the percentage of cells with accumulations. This was accomplished by high-content imaging using the Opera Phenix. The line with the most accumulations was chosen for subsequent assays.

Neuroblastoma cells were cultured in 50% EMEM (ATCC 30-2003), 50% Ham’s F-12 (Invitrogen 11765-054), and 10% FBS, tetracycline free (Clonetech 631101). Media were supplemented with 1% Glutamax (Invitrogen 35050-061), 1% MEM non-essential amino acids (Invitrogen 1140-050), and penicillin-streptomycin; 1 μg/ml puromycin was used in the stock media for αSyn 3K-GFP selection.

αSyn 3K-GFP accumulations were assessed after 48-h induction with doxycycline (2 μg/ml) by counting the number of cells that were positive, giving a % of cells that harbored accumulations. This was accomplished with live imaging using PerkinElmer Opera Phenix high-content screening system.

### iPSC neuron culture

Human iPSC source was a Lymphoblastoid Cell Line ND07189 obtained from Coriell Cell Repository. The donor is a non-Hispanic/Latino, white male (46 XY). iPSCs were generated using the Epi5 Episomal iPSC Reprogramming kit (ThermoFisher Scientific). iPSCs were confirmed to be (1) grossly karyotypically normal (46 XY); (2) pluripotent; (3) myoplasma and human pathogen-free (except for EBV+). Neurons were differentiated from 7189L-NGN2-H2 iPSCs which carry a tet-inducible NGN2 expression cassette targeted into the AAVS1 safe harbor locus. Three days after doxycycline treatment to induce NGN2 expression and differentiation, neurons were frozen.

Previously frozen NGN2 neurons were partially thawed in 37°C water bath. The exterior of vial was sterilized with 70% EtOH, then placed inside the TC hood. Cells were transferred to sterile 50-ml tubes, and slowly resuspended with 5 ml of room temperature NB/B27 plating media [Neurobasal:DMEM/F12 (1:1 vol) + 200 μm ascorbic acid + 1 μm dbcAMP + 10 ng/ml BDNF + 10 ng/ml GDNF + 1× pen/strep + 1 μg/ml doxycycline + CultureOne]. Cells were spun down at 800 rpm for 4 min at room temperature, and cell pellet was gently resuspended in NB/B27 plating media. Cells were counted on Vi-Cell and plated on PDL-coated microplates. To minimize edge effect, microplates were left for 30 min inside the TC hood to allow cells to attach, before placing inside the 37°C/CO_2_ TC incubator. Doxycycline treatment continued until DIV7, at which point half the media were exchanged with [Neurobasal:DMEM/F12 (1:1 vol) + 200 μm ascorbic acid + 1 μm dbcAMP + 20 ng/ml BDNF + 20 ng/ml GDNF + 1× pen/strep].

SCD inhibitor, AAV, and or OLA treatments began on DIV7 and DIV21 for early and late cultures, respectively.

### siRNA treatment

siRNAs were procured from Integrated DNA Technologies: hs.Ri.SCD.13.1, hs.Ri.SCD.13.2, hs.Ri.SCD.13.3, hs.Ri.SCD5.13.1, hs.Ri.SCD5.13.2, hs.Ri.SCD5.13.3. Three siRNAs were pooled in equal amounts for each target. siRNAs were delivered to neuroblastoma cells in Opti-MEM (Invitrogen) using Lipofectamine RNAiMAX (Invitrogen, 13778). siRNA treatments were given 24 h before doxycycline induction.

### Cell viability measurements

Cytotoxicity in neuroblastoma M17 αSyn 3K-GFP model was assessed by caspase 3 and 7 activity via a commercial kit (Promega, G8090). Before lysis and caspase measurement cell counts were taken by staining with Hoechst and Opera Phenix high-content imaging. Caspase activity was normalized to cell counts. Viability in rodent cortical neurons and in NGN2 neurons was assessed by CellTiter-Glo (Promega, G7571). In addition, neuronal and astrocytic viability in rat cortical neurons was determined after fixation, permeabilization (0.1% Triton X-100 for 20 min), and staining with MAP2 (Abcam, ab92434) and GFAP (Abcam, ab7260) by neuron and astrocyte counting in the Opera Phenix high-content imaging system. MAP2 and GFAP were visualized by Alexa Fluor 647 and 488 secondary antibodies.

### qRT-PCR

mRNA isolation was conducted one of two ways. For primary and iPSC neurons, the Cells-to-CT kit was used for mRNA isolation and cDNA synthesis (TaqMan Fast Advanced Cells-to-CT kit, A35374). For neuroblastoma cells, the Cells-to-CT kit was used or the combination of the RNeasy Plus Micro kit (QIAGEN, 74134) with iScript Reverse Transcription Supermix for RT-qPCR (Bio-Rad, 1708840) for mRNA isolation and cDNA synthesis. qRT-PCR was then performed using the TaqMan Fast Advanced Master Mix (ThermoFisher Scientific, 4444556) with QuantStudio 7 Pro Real-Time PCR System.

For qRT-PCR, target genes were normalized and multiplexed with HPRT gene expression (neuroblastoma and iPSC neurons) or with ACTB (rat primary neurons). Reference genes used VIC and target genes FAM dye signal.

TaqMan primer list: HPRT1, Hs02800695_m1; SCD, Hs01682761_m1; SCD5, Hs00227692_m1; SNCA, Hs00240906_m1; ACTB, Rn00667869_m1; SCD1, Rn06152614_s1; and SNCA, Rn01425140_m1.

### Human tissue

Frozen postmortem amygdala tissues of a non-demented control donors (female, 70 years of age) and a donor with clinical diagnosis of PD (male, 87 years of age, Braak stage 6) were obtained from the Netherlands Brain Bank (NBB; Netherlands Institute for Neuroscience, Amsterdam, open access; www.brainbank.nl). Written informed consent for the use of the samples for research purposes and clinical information of donors was obtained by the NBB.

### Western blotting and dot blot analysis

Cells were lysed in RIPA buffer with protease and phosphatase inhibitors. Lysates were protein normalized using BCA assay. Where indicated proteins were sequentially extracted from tissue or cells in high salt (750 mm), 1% Triton X-100 buffer and 1% SDS buffer as described previously (Weihofen et al., 2019). For Western blotting, proteins were then resolved on Bolt gels (4–12%) under reducing conditions using MES SDS running buffer system, followed by transfer to PVDF membrane with the iBlot 2 Gel Transfer system. For dot blot analysis, total extracts were spotted onto 0.45-μm nitrocellulose membranes (normalized for total αSyn expression). Membranes were blocked in either 5% milk in TBS (0.05% Tween) or LICOR blocking buffer (0.05% Tween 20) and probed with primary antibodies diluted in respective blocking buffer. Bound antibodies were detected by enhanced chemiluminescence or LICOR infrared imaging system.

### Statistical analysis

Analyses were performed with GraphPad Prism 9. Significant level was defined as *p* < 0.05. Intergroup differences were tested using two-tailed *t* test, one-way ANOVA or two-way ANOVA. Multicomparisons were corrected with Dunnett’s *post hoc* test when comparing three or more groups (for details, see Extended Data Fig. 1-2). Data were displayed as boxplot or line charts showing error bars with SDs. Statistical analyses for RNA-Seq data are described in its own section above.

## Results

**Figure 1.**
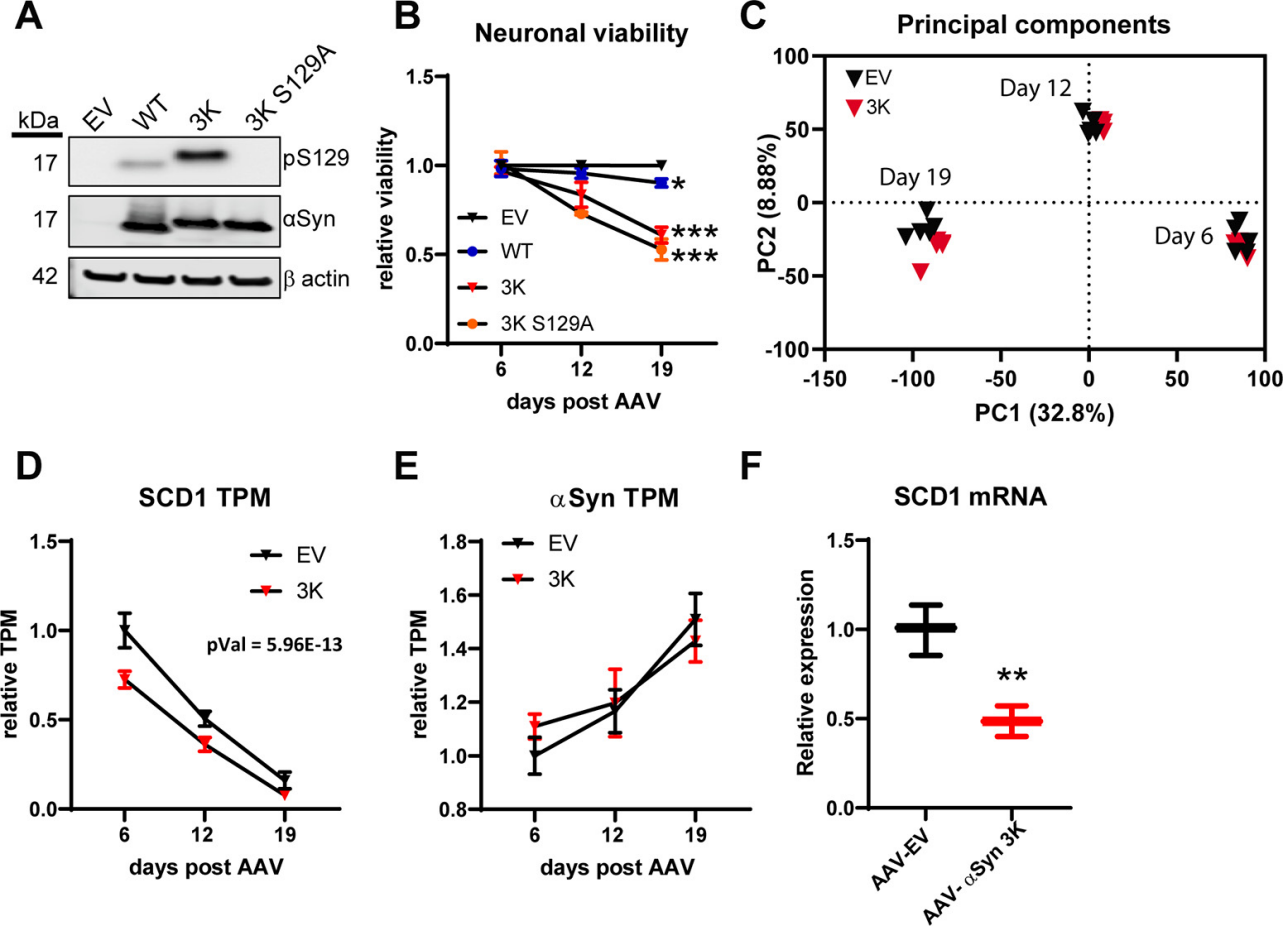
RNA-Seq identifies SCD as a αSyn 3K neurotoxicity regulated gene in rat cortical neuron cultures. ***A***, Western blotting showing equivalent expression of αSyn WT, αSyn 3K, or αSyn 3K S129 phosphorylation mutant (S129A) in rat cortical neuron cultures on respective AAV transduction. ***B***, Viability assessed by ATP levels (CellTiter-Glo) in rat cortical neuron cultures under respective AAV9 transduction, two-way ANOVA with Dunnett’s multiple test correction. Dose-dependent and time-dependent toxicity analysis in mouse and rat cortical neuron cultures are shown in Extended Data Figure 1-1. ***C***, Principal component analysis of RNA-Seq data from rat cortical neurons with AAV9-EV or AAV9-αSyn 3K (6, 12, and 19 d after AAV9 transduction). ***D***, Relative transcripts per million of SCD1 transcripts in AAV9-EV and AAV9-αSyn 3K samples. ***E***, Relative transcripts per million of endogenous rat SNCA (αSyn) transcripts in AAV9-EV and AAV9-αSyn 3K samples. ***F***, qPCR confirmation of SCD1 transcript suppression from AAV9-αSyn 3K in rat cortical neuron cultures 12 d after AAV9 transduction, two tailed *t* test. Data displayed as boxplots or as line charts showing error bars with SD; *p < 0.05, **p < 0.01, ***p < 0.001. Extended Data Figure 1-2 displays additional information on statistical tests applied in present study.

To reveal mechanistic insights into αSyn neurotoxicity, we performed gene expression profiling on neurons overexpressing cytotoxic αSyn. To do so, we first overexpressed human wild-type αSyn (αSyn WT) and αSyn 3K in rat primary cortical neuron cultures using AAV9. Equivalent overexpression was confirmed by Western blotting for αSyn WT and 3K (Fig. 1*A*). Cell viability was assessed by measuring ATP levels and was significantly reduced by ∼40% 19 d after AAV9 αSyn 3K compared with EV AAV9 transduction (Fig. 1*B*). A clear trend toward cytotoxicity was observed after 12 but not 6 d with αSyn 3K (Fig. 1*B*). In contrast, only minor or no cytotoxicity was observed with αSyn WT in this system (Fig. 1*B*). Dose-dependent and time-dependent cytotoxicity was observed in rat and mouse cortical neuron cultures, where mouse cultures showed greater sensitivity to αSyn 3K stress (Extended Data Fig. 1-1). Of note, we found αSyn 3K to be hyperphosphorylated at S129 in comparison to αSyn WT, and mutation of the phosphorylation site (S129A) did not reduce cytotoxicity (Fig. 1*A*,*B*). This suggests that phosphorylation at S129 is not required for the cytotoxic properties of αSyn 3K.

10.1523/ENEURO.0166-21.2021.f1-1 Extended Data Figure 1-1 αSyn WT versus 3K toxicity in rat and mouse cortical neuron cultures. Time (***A***) and dose (***B***; MOI of 62.5K, 125K, or 250K)-dependent toxicity measured by CellTiter-Glo in primary rat cortical neuron cultures treated with AAV9-EV or AAV9-αSyn 3K. ***C***, ***D***, Same as ***A***, ***B*** except carried out in mouse cortical neuron cultures. Time-dependent plots ***A***, ***C*** were done with AAV9 MOI of 250K. Dose-dependent plots ***B***, ***D*** were assessed 19 d after AAV9 transduction. Note that AAV9-αSyn 3K transduction is always more toxic than WT or EV control. ***E***, Relative transcripts per million of SCD2 transcripts in AAV9-EV and AAV9-αSyn 3K samples. Data displayed as line charts showing error bars with SD. Download Figure 1-1, TIF file.

**Figure 2.**
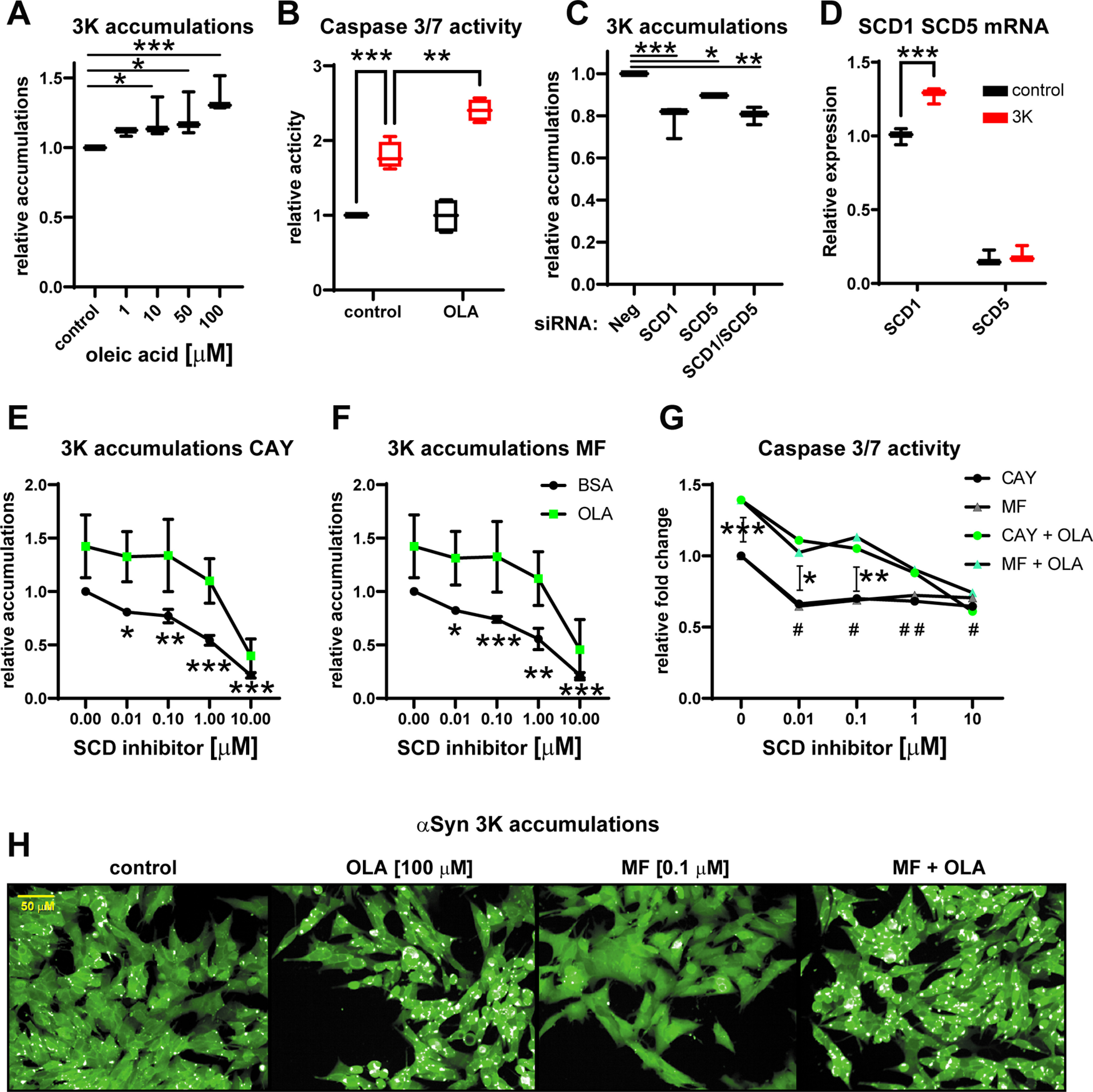
Inhibition of SCD in αSyn 3K-GFP neuroblastoma model reduces accumulations and cytotoxicity. ***A***, Dose-dependent increase in αSyn 3K-GFP accumulations from 48-h OLA treatment (1–100 μm). ***B***, 48-h induction of αSyn 3K-GFP leads to caspase 3/7 activation and 100 μm OLA treatment further increases it, whereas SCD1 siRNA reduces it (Extended Data Fig. 2-1*A*) ***C***, 5 nm siRNA knock-down of SCD1 or SCD5 for 48 h decreases the number of cells with accumulations. Please see Extended Data Figure 2-1*B–E* for further siRNA knock-down characterization. ***D***, mRNA levels of SCD1 and SCD5 in our neuroblastoma model showing SCD5 has ∼80% less expression than SCD1 and that accumulations can increase SCD1 levels. ***E***, Relative fraction of cells with accumulations under increasing concentration of CAY for 48 h with and without 100 μm OLA. ***F***, Relative fraction of cells with accumulations under increasing concentration of MF for 48 h with and without 100 μm OLA. Both prophylactic and therapeutic treatment paradigms (Extended Data Fig. 2-1*F*) reduce accumulations. ***G***, Relative αSyn 3K-GFP accumulation-induced caspase activity with increasing concentration of CAY or MF with and without 100 μm OLA for 48 h (* denotes comparison between non-OLA and OLA treatments, # denotes respective inhibitor concentration vs DMSO control). Note that OLA does not reestablish the αSyn 3K accumulation-induced caspase activity under SCD inhibition ≥1 μm. ***H***, Representative high-content images of αSyn 3K-GFP neuroblastoma model with induced αSyn 3K accumulations showing OLA-induced increase, SCD inhibitor-induced decrease, and OLA rescue of accumulations from SCD inhibition. Additional αSyn 3K accumulation characterization including comparison to LBs is shown in Extended Data Figure 2-2. One-way or two-way ANOVA run with Dunnett’s multiple test correction, data displayed as boxplots or as line charts showing error bars with SD, *p < 0.05, **p < 0.01, ***p < 0.001. All plots *n* ≥ 3 independent experiments.

10.1523/ENEURO.0166-21.2021.f1-2 Extended Data Figure 1-2 Statistical table. Download Figure 1-2, XLS file.

Having found αSyn 3K to elicit robust toxicity in neuron cultures we then performed RNA-Seq on rat cortical neuron cultures treated with AAV9 αSyn 3K or EV with isolations at 6, 12, and 19 d after AAV transduction. Principal component analysis of the gene expression profiling in our primary rat neuron cultures showed the greatest transcriptional changes between different culture ages (6 vs 12 vs 19 d after AAV9; Fig. 1*C*). Furthermore, the expression of endogenous αSyn increases in the neuron cultures through time (Fig. 1*E*), which has been reported in primary neuron maturation previously (Courte et al., 2020). The PCA and αSyn expression data combined suggest that our primary cultures are maturating *in vitro*. Separation of EV and αSyn 3K groups began at 12 d after transduction (Fig. 1*E*). Table 1 shows a list with the top ten significantly changed genes when including all three time points. We focused on gene hits found in all three time points to ensure robustness of the hits and to uncover pathologic changes occurring before obvious cytotoxicity was observed. Many of these hits were lipid regulatory genes, and indeed pathway analysis showed enrichment in lipid processes and fatty acid metabolism (Extended Data Table 1-2). Among these top hits was SCD1, which was downregulated by αSyn 3K (Fig. 1*D*). We also confirmed SCD1 downregulation by αSyn 3K by qPCR (Fig. 1*F*). The SCD2 isoform, which has high expression in the rodent brain, was also down regulated by αSyn 3K (Extended Data Fig. 1-1*E*; Kaestner et al., 1989).

**Table 1:** Top 10 differentially regulated genes by αSyn 3K in primary neuron cultures

Rank	Gene name	log2FC	Combined FDR *p* value
1	Lpcat1	0.84	2.64E-86
2	Rnf145	0.61	5.14E-35
3	Rgs4	0.44	2.02E-17
4	Scd1	−0.67	5.96E-13
5	Fam102b	0.84	8.20E-12
6	Acsbg1	−0.25	1.34E-10
7	Snn	0.45	5.31E-10
8	Psat1	−0.29	1.34E-09
9	Rgcc	−0.4	1.07E-08
10	Pcdh8	0.26	2.01E-08

A meta-analysis was performed to identify the top differentially regulated genes at 6, 12, and 19 d after AAV9 transduction time points. These are the top 10 genes from this meta-analysis (see Extended Data Table 1-1 for the full results, and Extended Data Table 1-2 for pathway analysis); *p* values are corrected for false discovery (for details, see Materials and Methods).

10.1523/ENEURO.0166-21.2021.t1-1 Extended Data Table 1-1 Meta-analysis RNAseq full data set. Download Table 1-1, XLS file.

10.1523/ENEURO.0166-21.2021.t1-2 Extended Data Table 1-2 Reactome Pathway analysis. Download Table 1-2, XLS file.

Next, we wanted to assess the effects of SCD manipulation on αSyn accumulation formation and cytotoxicity. To do so, we developed a M17 human neuroblastoma cell model with doxycycline-inducible αSyn 3K-GFP expression. Similar αSyn 3K models, which display cytotoxicity and form lipid rich αSyn accumulations that are a potential early pathogenic step toward LBs, had previously been developed in the same neuroblastoma cell line (Dettmer et al., 2017; Imberdis et al., 2019; Terry-Kantor et al., 2020). To highlight differences between LBs and αSyn 3K accumulations, we show that pS129 αSyn is mostly soluble in our neuroblastoma αSyn 3K model while it is almost exclusively Triton X-100 insoluble and displays a ladder-like pattern on SDS PAGE in PD patient brain (Extended Data Fig. 2-2*A*,*B*). Similarly, no signal for oligomeric/fibrillar αSyn was detected in a dot blot analysis with αSyn 3K neuroblastoma extract (Extended Data Fig. 2-2*C*). For quantifying αSyn 3K-GFP accumulations we applied high-content imaging and used caspase 3/7 activation for assessing αSyn 3K-induced cytotoxicity. αSyn 3K accumulations were reduced by ∼80% if cells where fixed in presence of 1% Triton X-100, showing that accumulations are not composed of fibrillar αSyn (Extended Data Fig. 2-2*D*,*E*). We then recapitulated a dose-dependent increase in accumulation formation from treatment with the SCD product OLA (Fig. 2*A*,*H*; Fanning et al., 2019). We also found that the formation of accumulations coincides with increased caspase 3/7 activity and that OLA treatment can further increase it (Fig. 2*B*).

10.1523/ENEURO.0166-21.2021.f2-1 Extended Data Figure 2-1 siRNA knock-down of SCD characterization in αSyn 3K-GFP neuroblastoma model. ***A***, Relative caspase activation under 5 nm siRNA knock-down of SCD1 in 3K neuroblastoma model. Note the rescue of αSyn 3K-GFP accumulation induced toxicity and caspase activation with SCD1 knock-down. ***B***, Relative expression of SCD1 under 5 nm siRNA knock-down of SCD1 or SCD5. ***C***, Same as ***B*** except SCD5 expression. Note that knock-down of SCD1 increases expression of SCD5. ***D***, Representative Licor Western blotting depicting αSyn 3K-GFP, βactin, and SCD1 protein levels from samples treated as in ***A–C***. siRNA treatment was performed 6 h before induction of αSyn 3K, all assessments were done 48 h after induction. ***E***, Quantification of αSyn 3K-GFP protein levels as depicted in ***D***, note that siRNA knock-down does not alter 3K protein levels. ***F***, Accumulations were induced for 2 d before 1 d of SCD inhibitor (CAY or MF) treatment, *n* = 3 technical replicates. One-way or two-way ANOVA run with Dunnett’s multiple test correction, all data displayed as boxplots, *p < 0.05, **p < 0.01, ***p < 0.001. All plots *n* ≥ 3 independent experiments unless indicated otherwise. Download Figure 2-1, TIF file.

10.1523/ENEURO.0166-21.2021.f2-2 Extended Data Figure 2-2 Triton X-100 solubility of αSyn 3K-GFP accumulations. Sequential extraction with 750 mm NaCl (HS), 1% Triton TX-100 (TX soluble), and 1% SDS (TX insoluble) buffer in equal volume of (***A***) amygdala from control donor and donor with PD and (***B***) M17 cells overexpressing αSyn 3K GFP under Dox-inducible promoter. Western blotting for αSyn (MJFR1 at 1:1000 for ***A*** and 1:50,000 for ***B***) and pSer129 αSyn (EP1536Y at 1:1000 for ***A*** and 1:25,000 for ***B***). ***C***, Dot blot analysis probing total extracts of M17 cells overexpressing αSyn 3K GFP (+/–Dox), control and PD extracts with total αSyn (Syn-1 1:1000) and oligomeric/fibrillar αSyn antibody (MJFR 14-6-4-2 1:10,000). ***D***, Representative images of samples from ***E*. *E***, Relative accumulation levels in 4% PFA fixed neuroblastoma cells with and without 1% Triton X-100 permeabilization, *n* = 18 technical replicates, Student’s *t* test; *p < 0.05, **p < 0.01, ***p < 0.001. Download Figure 2-2, TIF file.

Humans have two SCD isoforms (SCD1 and SCD5), and we tested whether their suppression influenced accumulation formation with siRNA knock-down. mRNA reduction of both isoforms was robust (∼90%) and in the case of SCD1 led to a near complete loss of protein (Extended Data Fig. 2-1*B–D*). No antibody for measuring SCD5 protein levels is readily available. siRNA knock-down of both SCD1 and SCD5 led to a suppression of accumulations, albeit to a lesser degree by SCD5 knock-down (∼20% vs 10% suppression; Fig. 2*C*). Of note, SCD5 is expressed ∼80% less than SCD1 in our neuroblastoma model but still influenced accumulation formation (Fig. 2*D*). SCD1 knock-down also rescued αSyn 3K-GFP accumulation-induced toxicity as assessed by caspase 3/7 activity (Extended Data Fig. 2-1*A*). We also recapitulated a dose-dependent decrease in accumulations from the SCD inhibitors CAY and MF (Fig. 2*E*,*F*,*H*). SCD inhibition suppressed accumulations prophylactically and also therapeutically as when inhibitors were given after accumulation formation (Extended Data Fig. 2-1*F*). The observed decreases in accumulations at higher concentrations were much larger compared with complete knock-down of SCD1 or SCD5 by siRNA. At lower concentrations, the reduction of accumulations from SCD inhibition was reestablished by OLA treatment (Fig. 2*E*,*F*,*H*). Moreover, SCD inhibitors also reduced αSyn 3K-induced caspase 3/7 activation, and OLA reestablished caspase 3/7 activity too at lower concentrations (Fig. 2*G*).

**Figure 3.**
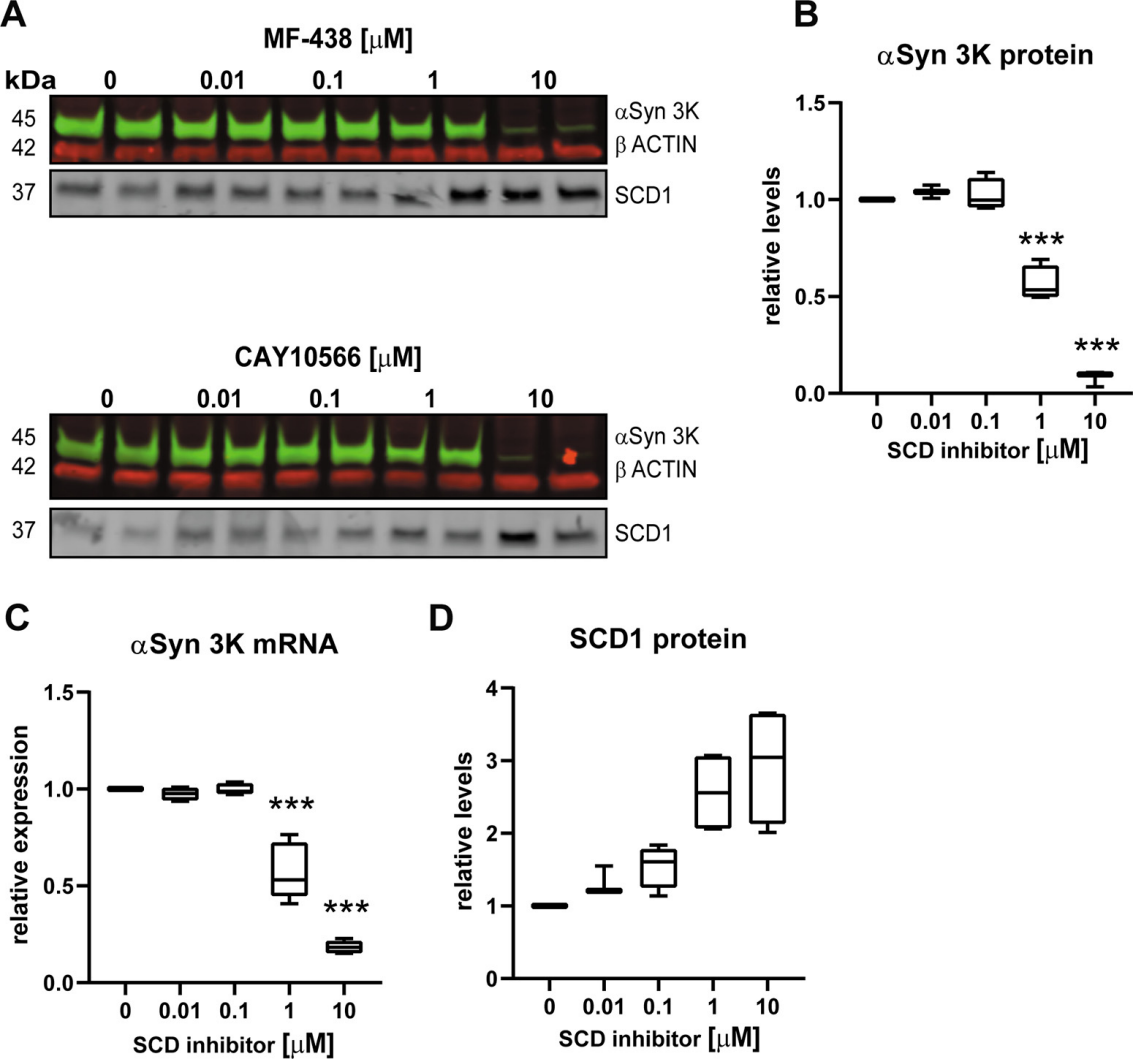
High SCD inhibitor concentrations suppress αSyn 3K-GFP mRNA and protein in neuroblastoma model. ***A***, Representative Licor western blot depicting αSyn 3K-GFP, βactin, and SCD1 protein levels from samples treated with SCD inhibitors (0.01 to 10 μM), αSyn 3K-GFP induction and SCD inhibitor treatment was 48 hours before protein isolation. ***B***, Quantification of αSyn 3K protein levels as in ***A***, CAY10566 and MF-438 data was equivalent and merged for this plot. ***C***, mRNA levels of αSyn 3K-GFP at equivalent time points and treatments as in ***C***. Note the drop in αSyn 3K protein and mRNA under SCD inhibition ≥ 1 μM. ***D***, Quantification of SCD1 protein levels under increasing concentrations of SCD inhibitor, note the dose dependent increase. Please see Extended Data Figure 3-1 for further SCD mRNA and protein characterization under inhibitor treatment. One-way ANOVA run with Dunnett’s multiple test correction, all data displayed as boxplots, *** *p* < 0.001. All plots n ≥ 3 independent experiments.

Interestingly, at higher concentrations of SCD inhibition (1 and 10 μm) we observed that OLA did not reestablish αSyn 3K-GFP-induced accumulations or caspase 3/7 activity (Fig. 2*E–G*), suggesting off-target effects. To explore this further we measured mRNA and protein levels of αSyn 3K-GFP with CAY and MF inhibition from 0.01 to 10 μm. We discovered that at ≥1 μm, there was a dose-dependent suppression of αSyn 3K-GFP mRNA and protein (Fig. 3*A–C*), 1 μm produced a ∼50% reduction and 10 μm a ∼90% reduction. In contrast to exogenous αSyn 3K-GFP, endogenous αSyn was unaffected by SCD inhibition (Extended Data Fig. 3-1*A*). Additionally, robust siRNA knock-down of SCD1 or SCD5 did not alter αSyn 3K-GFP protein levels, suggesting it is not a specific phenomenon related to SCD inhibition (Extended Data Fig. 2-1*D*,*E*). Overall, we validate that SCD inhibition lowers αSyn 3K accumulations and reduces αSyn 3K-induced cytotoxicity but add caution to any users of this type of αSyn 3K-GFP model (or similar overexpression system) as high inhibitor concentrations suppressed exogenous mRNA which led to off-mechanism (non-MUFA related) lowering of αSyn 3K accumulations.

10.1523/ENEURO.0166-21.2021.f3-1 Extended Data Figure 3-1 SCD1 mRNA compensatory upregulation in neurons and neuroblastoma cells under SCD inhibition. ***A***, Representative Licor Western blotting depicting endogenous SNCA (αSyn), βactin, and SCD1 protein levels from neuroblastoma cells, SCD inhibitor treatment was 48 h before protein isolation. ***B***, Relative SCD1 mRNA levels in neuroblastoma αSyn 3K-GFP model under 0.01 or 0.1 μm SCD inhibition ± 100 μm OLA (48-h treatments). ***C***, Relative SCD1 mRNA levels in primary rat cortical neuron cultures under 0.01 or 0.1 μm SCD inhibition ± 100 μm OLA (12-d treatments starting in DIV7 early cultures). Note that SCD1 mRNA increases under SCD inhibitor in both primary neurons and neuroblastoma cells and that this increase is reversed by OLA. Two-way ANOVA run with Dunnett’s multiple test correction, all data displayed as boxplots, *p < 0.05, **p < 0.01, ***p < 0.001. Download Figure 3-1, TIF file.

**Figure 4.**
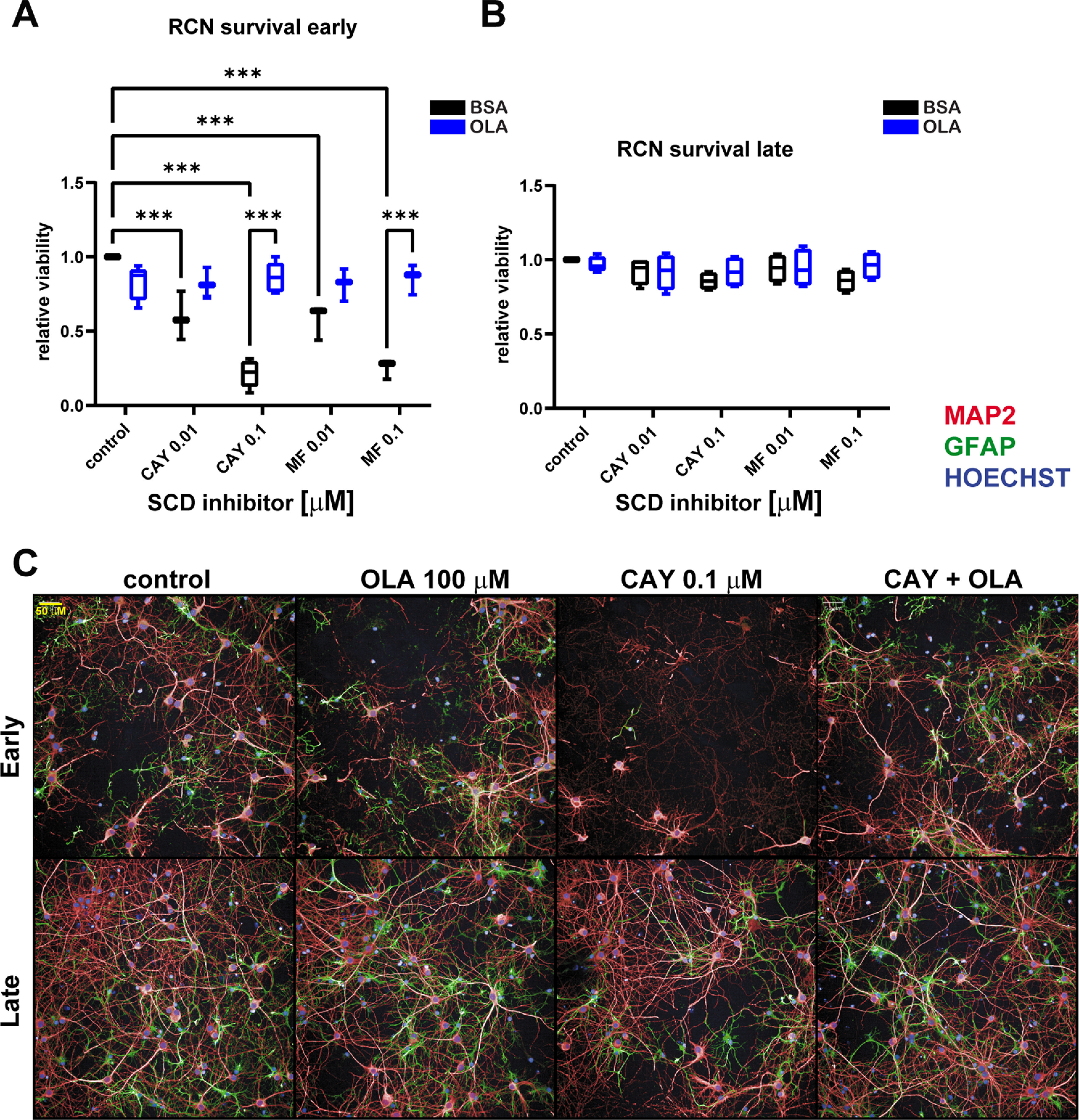
Early primary neuron cultures are sensitive to SCD inhibitor indued toxicity, while late cultures are resistant. ***A***, ***B***, CellTiter-Glo analysis on primary rat cortical neuron cultures after 12-d treatment ± SCD inhibitors (0.01 or 0.1 μm) ± OLA (100 μm) starting at DIV7 (***A***, early) or DIV18 (***B***, late). ***C***, Representative high-content images of both early and late cultures as in ***A***, ***B*** with OLA, CAY, or both CAY and OLA. Note the SCD inhibitor induced toxicity in early cultures that is rescued by OLA, while there is no toxicity in the late cultures. Two-way ANOVA run with Dunnett’s multiple test correction, all data displayed as boxplots, ***p < 0.001. All plots *n* ≥ 3 independent experiments.

**Figure 5.**
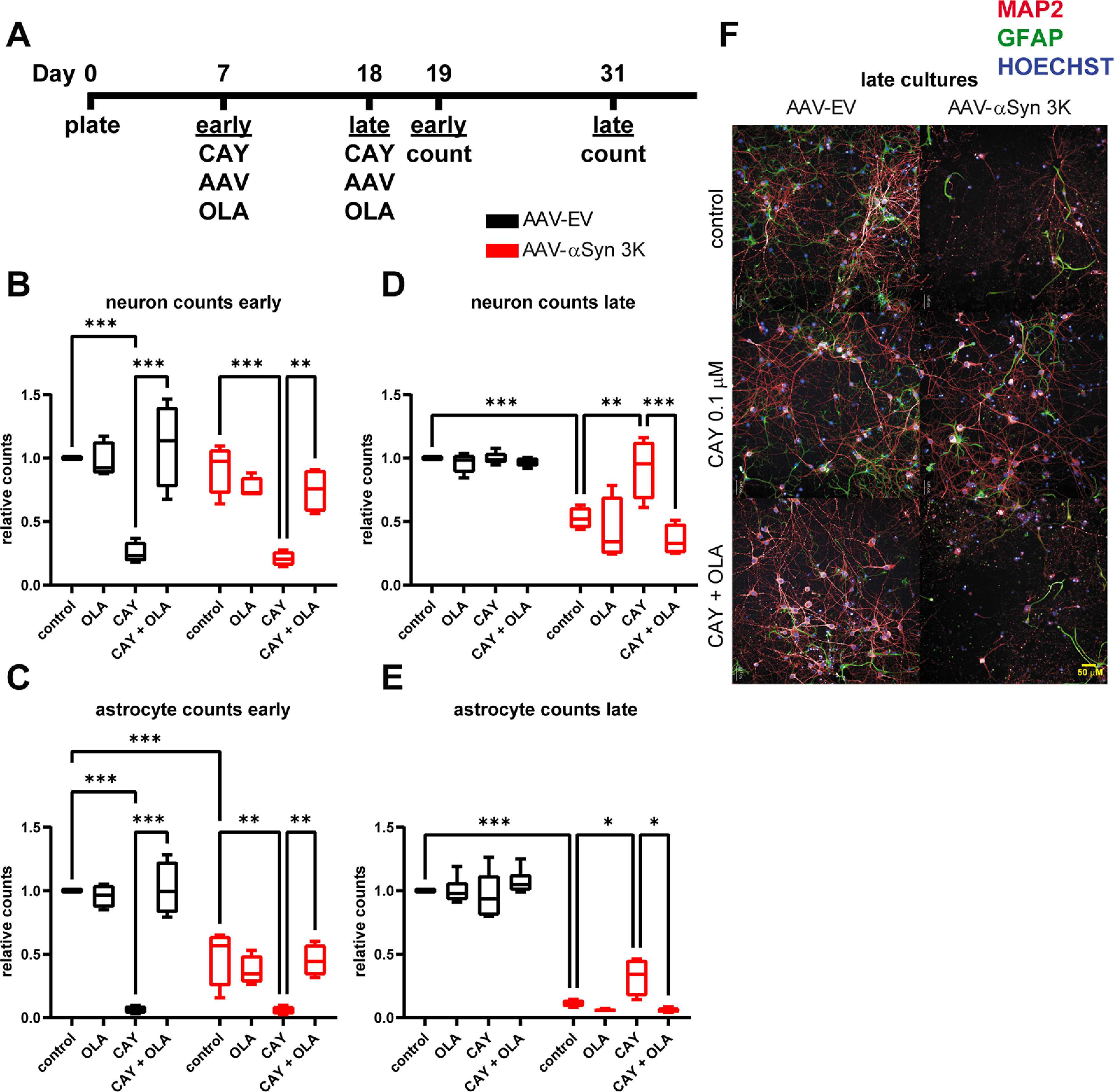
Late primary neuron cultures are protected from αSyn 3K toxicity by SCD inhibition (CAY). ***A***, Schematic of treatment schedule in early and late cultures. Primary rat cortical neuron cultures were treated for 12-d ± SCD inhibitors (0.01 or 0.1 μm) ± OLA (100 μm) + AAV9-EV or AAV9-αSyn 3K starting at DIV7 (early) or DIV18 (late). Neurons and astrocytes were counted by staining with MAP2 and GFAP markers, respectively. ***B***, Neuron counts from early primary cultures. ***C***, Neuron counts from late primary cultures. ***D***, Astrocyte counts from early cultures. ***E***, Astrocyte counts from late cultures. ***F***, Representative high-content images of late cultures as in ***B***, ***D***. Note that late cultures are rescued from αSyn 3K toxicity by SCD inhibition, which can be reversed by OLA treatment. Extended Data Figure 5-1 shows results with MF. Two-way ANOVA run with Dunnett’s multiple test correction, all data displayed boxplots, *p < 0.05, **p < 0.01, ***p < 0.001. All plots *n* ≥ 3 independent experiments.

Next, we wanted to assess the effects of SCD manipulation in primary neuron cultures. We treated 7 DIV cultures with 0.01 and 0.1 μm SCD inhibitors and assessed viability with ATP levels 12 d after treatment. We found that DIV7 cultures showed dose-dependent cytotoxicity from both CAY and MF compounds (Fig. 4*A*,*C*), 0.01 μm led to a ∼50% drop and 0.1 μm a ∼75% drop in viability. Importantly, the SCD inhibitor-induced toxicity was fully rescued by treatment with OLA, suggesting the cytotoxicity is on-mechanism. The finding of SCD inhibitor-induced toxicity in primary neurons was surprising as one study showed tolerance of SCD inhibitors on primary rat neurons (Fanning et al., 2019). SCD1 expression decreases overtime in these cultures suggesting that they become less dependent on SCD and OLA as they mature (Fig. 1*D*). To further explore SCD inhibitor toxicity we repeated the experiment starting at DIV18, hypothesizing more established cultures may have increased tolerance. Indeed, we found no significant SCD inhibitor-induced cytotoxicity in these late cultures (Fig. 4*B*,*C*). To confirm that neuron death was occurring in early DIV7 cultures with SCD inhibition, we repeated the viability experiment and counted neurons and astrocytes present in these cultures by staining with MAP2 and GFAP markers, respectively. We found that both neurons and astrocytes were dying from SCD inhibition to a similar degree (Fig. 5*B*,*C*). Both neuron and astrocyte populations were also rescued by OLA treatment. Overall, these data show that early (∼DIV7) primary cultures are susceptible to SCD inhibition toxicity but that late (∼DIV18) cultures are resistant.

10.1523/ENEURO.0166-21.2021.f5-1 Extended Data Figure 5-1 Late primary neuron cultures are protected from αSyn 3K toxicity by SCD inhibition (MF). ***A***, Neuron counts from early primary rat cortical neuron cultures treated with MF (0.01 or 0.1 μm) ± OLA (100 μm) + AAV9-EV or AAV9-αSyn 3K for 12 d. ***B***, Same as ***A*** except treatments starting in late cultures. ***C***, Astrocyte counts from early cultures. ***D***, Astrocyte counts from late cultures. Experimental paradigm for ***A–D*** is equivalent to Figure 5, with the addition of AAVs and high-content image counting with MAP2 and GFAP markers for neurons and astrocytes, respectively. ***E***, Representative high-content images of late cultures as in ***B***, ***D***. Note that late cultures are rescued from αSyn 3K toxicity by SCD inhibition, which can be reversed by OLA treatment. Two-way ANOVA run with Dunnett’s multiple test correction, all data displayed as boxplots, *p < 0.05, **p < 0.01, ***p < 0.001, ****p < 0.0001. All plots *n* ≥ 3 independent experiments. Download Figure 5-1, TIF file.

Having demonstrated that SCD inhibition toxicity was dependent on primary culture age (DIV7 vs DIV18), we then wanted to assess SCD inhibition effects on αSyn-induced cytotoxicity. We treated both early and late cultures with CAY, AAV9-αSyn 3K or EV, and OLA for 12 d and then counted neurons and astrocytes (Fig. 5*A*). Early cultures were more resistant to αSyn 3K stress overall (∼20% neuron loss vs ∼50% in late neurons) and did not receive any protection from SCD inhibition (as observed before SCD inhibition was toxic; Fig. 5*B*,*C*). Although late neuron cultures were more susceptible to αSyn 3K toxicity, they were rescued by SCD inhibition (Fig. 5*D*,*E*). Importantly, OLA treatment ablated the SCD inhibitor rescue of αSyn 3K toxicity in late neurons and astrocytes, suggesting the rescue was on-mechanism (Fig. 5*D–F*). OLA itself exacerbated neuron and astrocyte death under αSyn 3K stress in both early and late cultures, supporting its role in promoting αSyn pathology (Fig. 5*B–E*). Astrocytes showed similar trends to neurons where αSyn 3K toxicity was more severe in the late cultures (∼60% vs ∼90% astrocyte loss). Viability results with MF was equivalent to CAY (Extended Data Fig. 5-1). Taken together, these data demonstrate that the suppression of MUFAs through SCD inhibition can alleviate αSyn 3K stress in late primary neuron cultures but that early cultures receive no protection.

Notably, SCD inhibition by CAY or MF caused an increase in SCD1 mRNA and protein levels in our neuroblastoma model and our primary neuron cultures (Fig. 3; Extended Data Fig. 3-1). The increase in mRNA and protein levels following SCD inhibition was reversed by OLA treatment in both models, suggesting it is a compensatory mechanism. SCD upregulation on its inhibition by small molecules has been observed previously (Vincent et al., 2018). These data further suggest mechanism and robust SCD inhibition by CAY and MF in our *in vitro* models.

**Figure 6.**
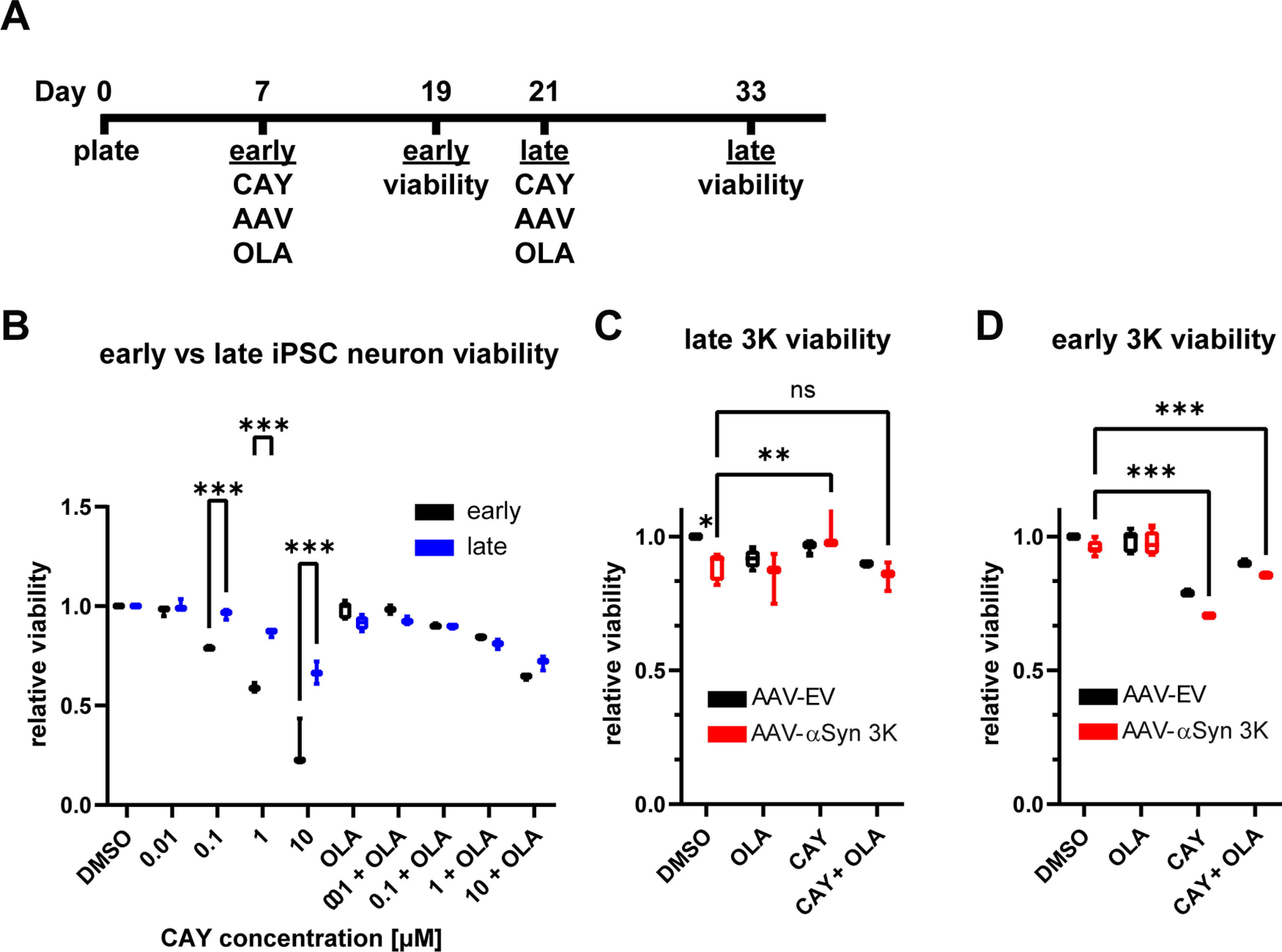
Late human iPSC neurons are protected from αSyn 3K toxicity by SCD inhibition (CAY) and are less sensitive to SCD inhibition toxicity. ***A***, Schematic of treatment schedule in early and late cultures. ***B***, CellTiter-Glo analysis on human iPSC neuron cultures after 12-d treatment ± CAY (0.01, 0.1, 1, or 10 μm) ± OLA (10 μm) starting at DIV7 (early) or DIV21 (late). ***C***, ***D***, Same as ***B*** but only with 0.1 μm CAY and with AAV9-EV or AAV9-αSyn 3K for 12-d in late (***C***) or early (***D***) cultures. Note the rescue of viability in late iPSC neuron cultures by 0.1 μm CAY (a concentration non-toxic to late neurons but toxic to early neurons). Extended Data Figure 6-1 shows results with MF, and Extended Data Figure 6-2 shows SCD single-cell expression in human substantia nigra. Two-way ANOVA run with Dunnett’s multiple test correction, all data displayed as boxplots, *p < 0.05, **p < 0.01, ***p < 0.001, ns = not significant. All plots *n* ≥ 3 independent experiments.

Human iPSC neurons have been shown to tolerate SCD inhibition and even be protected from αSyn toxicity (Vincent et al., 2018; Fanning et al., 2019; Nuber et al., 2021). Based on our differential response to SCD inhibition in our rat primary neuron cultures, we tested early versus late human iPSC neuron cultures under SCD inhibition. Early iPSC neuron cultures started treatment on DIV7 and late on DIV21. As before, SCD inhibitors and OLA treatment were done simultaneously, and cultures were assessed for ATP level viability 12 d later (Fig. 6*A*). Both early and late iPSC neuron cultures showed dose-dependent SCD inhibition-induced cytotoxicity. Similar to our primary neuron cultures, the late iPSC neuron cultures were more resistant to SCD inhibitor-induced toxicity. Late iPSC cultures were resistant up to 0.1 versus 0.01 μm for early cultures (Fig. 6*B*). For example, at 0.1 μm, early cultures had ∼20% less viability where late cultures had negligible loss on average. OLA rescued SCD inhibition toxicity in the early iPSC neuron cultures but appeared to have minimal effect in the older cultures.

10.1523/ENEURO.0166-21.2021.f6-1 Extended Data Figure 6-1 Late human iPSC neurons are protected from αSyn 3K toxicity by SCD inhibition (MF) and are less sensitive to SCD inhibitor toxicity. ***A***, CellTiter-Glo analysis on human iPSC neuron cultures after 12-d treatment ± MF (0.01, 0.1, 1, or 10 μm) ± OLA (10 μm) starting at DIV7 (early) or DIV21 (established). ***B***, ***C***, Same as ***A*** but only with 0.1 μm MF and with AAV9-EV or AAV9-αSyn 3K for 12 d in early (***B***) or late (***C***) cultures. Note the rescue of viability in late iPSC neuron cultures by 0.1 μm MF (a concentration non-toxic to establish neurons but toxic to early neurons). Two-way ANOVA run with Dunnett’s multiple test correction, all data displayed as boxplot, **p < 0.01, ***p < 0.001. All plots *n* ≥ 3 independent experiments. Download Figure 6-1, TIF file.

10.1523/ENEURO.0166-21.2021.f6-2 Extended Data Figure 6-2 SCD1 and SCD5 cell type-specific expression in human substantia nigra. ***A***, Relative expression of SCD1 and SCD5 from human substantia nigra single-cell RNA-Seq (Agarwal et al., 2020). Note that the expression of the SCD5 isoform is higher than SCD1 in all but the oligodendrocyte population. Astro, astrocytes; Endo, endothelial cells; MG, microglia; Neuron, neuron; OPC, oligodendrocyte precursor cells; Oligo, oligodendrocyte. Download Figure 6-2, TIF file.

Having established that iPSC human neurons display different sensitivity to SCD inhibition-induced toxicity based on *in vitro* age, we then compared early and late iPSC neuron cultures under 0.1 μm (a concentration that was non-toxic to late but toxic to early) under αSyn 3K stress. Even at higher AAV titers than that used in the primary neuron cultures (MOI 250K vs 80K), we only observed between 5% and 20% ATP loss in the iPSC neurons cultures with late cultures appearing more sensitive to 3K stress. Despite the minimal αSyn 3K-indued neurotoxicity, 0.1 μm SCD inhibition could rescue viability in late iPSC neuron cultures (Fig. 6*C*). OLA treatment reversed this rescue, suggesting on-mechanism protection. As expected, because of their toxicity, SCD inhibitors exacerbated αSyn 3K-induced toxicity in early iPSC neurons (Fig. 6*D*). Viability results with MF were equivalent to CAY (Extended Data Fig. 6-1). These data demonstrate that the differential response, based on *in vitro* age, to SCD inhibition-induced toxicity also occurs in human neurons and that non-toxic concentrations can protect neurons from αSyn stress.

## Discussion

We report that a significant factor in primary neuronal susceptibility to toxicity induced by SCD inhibition is their maturity or *in vitro* age. Our data suggest that there is early dependence on MUFAs and OLA that reduces with culture maturation. This is supported by our finding of early neuron and astrocyte populations being susceptible to SCD inhibition-induced toxicity that can be rescued by OLA treatment (Figs. 4*A*, 5*B*,*C*). Late neurons and astrocytes display no significant susceptibility to SCD inhibition toxicity (Figs. 4*B*, 5*D*,*E*), presumably because they are less reliant on MUFA synthesis and OLA. Further supporting this differential requirement of MUFAs is that the expression of SCD1 reduces through time in primary neuron cultures, dropping by ∼50% every 6 d between early and late cultures (Fig. 1*D*). Furthermore, SCD is a target of interest in cancer because it is upregulated because of high lipogenic and MUFA demand (from increased metabolic activity and division; Tracz-Gaszewska and Dobrzyn, 2019). A similar mechanism may be at play in early neuron cultures that also have a high metabolic demand and require OLA for synaptic development and neurotrophic support (Medina and Tabernero, 2002; Polo-Hernández et al., 2014) . Any investigator looking to use SCD inhibitors without toxicity side effects in primary neuron models should establish when they no longer induce significant cytotoxicity or when OLA is no longer critical for their survival.

SCD inhibition, when not toxic, rescues αSyn 3K toxicity in primary cultures which can be reversed by OLA treatment (Fig. 5*D*). OLA treatment itself exacerbated neuron and astrocyte death in our late cultures under αSyn 3K stress (Fig. 5*D*,*E*). This demonstrates suppression of MUFAs and OLA are key to SCD inhibition-induced protection. OLA levels have specifically been found to be upregulated by excess αSyn and to exacerbate αSyn stress (Fanning et al., 2019). The down regulation of SCD1 and SCD2 (Fig. 1*D*; Extended Data Fig. 1-1*E*) under αSyn 3K stress in primary cultures is likely a compensatory process to lower MUFAs and OLA, where significant SCD inhibition accomplishes this and provides protection (as with CAY and MF SCD inhibitors). Conversely, SCD1 was upregulated in our αSyn 3K neuroblastoma model (Fig. 2*D*). A transformed cell line likely processes αSyn 3K stress differently than primary cultures. In either model, the inhibition of SCD and MUFAs is beneficial.

Astrocytes in both the early and late primary cultures were more susceptible to αSyn 3K stress than neurons, this is clear in the early cultures where most death is in the astrocyte population (Fig. 5*A*,*B*). OLA synthesized by astrocytes has been shown to be important for neurotrophic support and synaptic development (Medina and Tabernero, 2002; Polo-Hernández et al., 2014). Astrocytes are also reported to have enhanced local translation of SCD and other fatty acid synthesis genes in their peripheral processes (Sakers et al., 2017). It is possible that astrocyte death occurs first from SCD inhibition and promotes neuron death from the loss of OLA and neurotrophic support in early cultures. Mature neuron death from αSyn 3K stress may similarly be promoted by the dysfunction and death of the astrocyte population. This is not without precedent as, when present, astrocytes can protect dopaminergic neurons from αSyn spreading and aggregation (Tsunemi et al., 2020). Furthermore, it has been shown that excess αSyn increases OLA, triglycerides, and their storage in lipid droplets in neurons (Fanning et al., 2019). Lipid droplets can sequester the build-up of fatty acids, however, neurons generally do not make lipid droplets where astrocytes have an enhanced ability for droplet formation and fatty acid mitigation (Bélanger and Magistretti, 2009). In fact, astrocytes have been shown to protect neurons from toxic fatty acid build-up with detoxification and consumption through Β-oxidation after their delivery from neurons (Ioannou et al., 2019). The interplay of astrocytes and neurons under αSyn 3K stress should be explored further. Additionally, the αSyn 3K model may be useful in the study of synucleinopathies with glial focused pathology such as multiple system atrophy.

Our results with human iPSC neuron cultures were similar to that of our primary rat neuron cultures. Older iPSC neuron cultures were more resistant to SCD inhibition-induced toxicity but did show some sensitivity (Fig. 6*B*) in contrast to late primary rat neuron cultures which displayed no significant toxicity at the same concentrations. One possible explanation is the that the primary rat neuron cultures are mixed and contain glia, where astrocytes and potentially microglia exert a protective effect. Another being the different SCD isoforms that are present in rodents versus humans, which is discussed below. Additionally, toleration of SCD inhibition has been shown in human iPSC neurons previously but it is unclear at what DIV inhibitors were administered (Vincent et al., 2018; Fanning et al., 2019; Nuber et al., 2021). Presumably in these studies SCD inhibitors were given in primary or iPSC neuron cultures that were matured sufficiently where MUFAs are no longer required for development and survival. To our knowledge this is the first-time showing iPSC neurons are protected specifically from αSyn 3K stress with non-toxic concentrations of SCD inhibitors. Researchers must consider culture age carefully when designing experimental paradigms with SCD inhibitors with *in vitro* iPSC neuron cultures.

Our independently generated αSyn 3K-GFP accumulation forming cell line confirmed many published results. However, our model showed that high inhibitor concentrations (≥1 μm) suppressed the expression and protein levels of αSyn 3K-GFP in an off-target manner (Fig. 3). In a similar neuroblastoma model it was found that αSyn 3K protein levels were unchanged up to 10 μm SCD inhibitor concentrations, although the CAY inhibitor showed a trend of protein lowering (Imberdis et al., 2019). It is important to note that the IC50s of CAY and MF are ∼0.005–0.01 μm in cell culture (Liu et al., 2007; Uto, 2016) and much higher concentrations are not significantly blocking SCD activity further. Moreover, maximal SCD inhibition at 0.1 μm and robust siRNA knock-down of SCD1 both produced ∼25% reduction in accumulations (Fig. 2). Together this suggests that maximal on target suppression of accumulations by SCD inhibition is ∼25%. Interestingly, a 25% reduction in accumulations fully rescued caspase 3/7 activity, suggesting a threshold for accumulation-induced cytotoxicity. Thus, there may be a smaller true effect of MUFA down regulation (through SCD inhibition) on αSyn 3K accumulation formation than previously reported. It is also possible our model’s αSyn 3K-GFP expression may be more sensitive to experimental perturbations because of expression construct design or potential parental cell line differences. Despite the issues with high inhibitor concentrations, we found similar results in the suppression of αSyn 3K accumulation formation and cytotoxicity with SCD inhibition in our neuroblastoma model.

Rats have two SCD isoforms (SCD1 and SCD2) which share ∼87% sequence homology. In cell culture SCD1 and SCD2 are both inhibited by CAY (Masuda et al., 2012). Taken together, it is likely that both isoforms were blocked by SCD inhibitors in our primary rat neuron culture experiments. Human SCD1 shares ∼85% sequence homology with all rodent SCD isoforms. But the second human SCD isoform, SCD5, is unique to primates, has limited homology to other isoforms, and has high expression in brain tissue (Wang et al., 2005). We examined published single-cell RNA-Seq data from the substantia nigra region in humans (Agarwal et al., 2020) and found SCD5 to be overall higher in human neurons and astrocytes (Extended Data Fig. 6-2), although SCD1 is higher in oligodendrocytes. Yumanity’s SCD inhibitor compound YTX-7739, currently in clinical development, targets both SCD1 and SCD5 (Cross, 2018). We believe targeting SCD5 is a good strategy to inhibit SCD activity more effectively in the brain and not elsewhere in the body. This is important because of the connection between SCD and MUFAs to proper immune function and other potential negative side effects of broad SCD inhibition (Meingassner et al., 2013; Zhou et al., 2021).

Out of the top 10 hits in our αSyn 3K toxicity gene expression profiling study, three genes have been previously linked to PD, namely SCD, regulators of G-protein signaling (RGS4) and acyl-CoA synthetase bubblegum family member 1 (ACSBG1). Inhibitors of RGS4 have been shown to reverse D2 antagonist-induced bradykinesia in rats (Blazer et al., 2015), generating interest in their use as novel therapeutics for PD. An RNAi screen discovered that ACSBG1 regulates αSyn levels, which was confirmed in human neurons and mouse brain tissue (Rousseaux et al., 2018). Many of the transcriptional profiling hits were lipid regulatory genes and pathway analysis indeed found several lipid metabolic process enrichments (Extended Data Table 1-2). For instance, sterol regulatory element binding factor 1 (SREBF1) is a transcription factor that regulates SCD and other lipogenic genes. SREBF1 has been identified in a PD genome wide association study and a functional screen for PD (Do et al., 2011; Ivatt et al., 2014). Overall, our transcriptional profiling supports the interconnection between αSyn pathology, MUFAs, and lipids more generally. Investigations of our most significant hit LPCAT1, which is also involved in lipid metabolism, is under way. The fact that many of the top gene hits are connected to PD supports the pursuit of investigating LPCAT1 and other novel hits previously unconnected to PD and supports the utility of the αSyn 3K model.

Overall, our study supports the use of SCD inhibitors in PD to ameliorate αSyn pathology and neurotoxicity. We demonstrate differential SCD inhibition toxicity based on neuron culture age which must be considered when researching SCD *in vitro* and has possible clinical implications. It is also important to note that a SCD inhibitor has been assessed in a preclinical mouse model, where it did not induce neurodegeneration and in fact protected from WT and 3K-induced neuron loss (Nuber et al., 2021). Since those who develop PD are overwhelmingly older adults, toxicity to developing or early neurons would not be a major issue; the principal concern is saving established neurons. However, the use of SCD inhibitors in younger people and children with more active neurogenesis should be carefully considered and investigated because of the concerns of neurotoxicity.

## Synthesis

Reviewing Editor: Mikko Airavaara, University of Helsinki Decisions are customarily a result of the Reviewing Editor and the peer reviewers coming together and discussing their recommendations until a consensus is reached. When revisions are invited, a fact-based synthesis statement explaining their decision and outlining what is needed to prepare a revision will be listed below. The following reviewer(s) agreed to reveal their identity: Nathalie Van Den Berge, Laura Volpicelli-Daley.

The authors provide further evidence that alterations in SCD gene are associated with asyn pathology and asyn-induced toxicity in PD, which further establishes the importance of investigating SCD as a potential therapeutic target in preclinical PD models. Many studies support that that inhibition of stearoyl-CoA desaturase SCD reduces alpha-synuclein pathology and neurotoxicity and that lowering MUFAs may be viable therapeutic approach trials.

The authors find that SCD inhibition (when not toxic) reduces formation of asyn inclusions and asyn-induced toxicity (caspase 3/7 activity) in their PD cell models. These effects were reversed by OLA treatment, demonstrating that suppression of MUFAs and OLA are key to SCD inhibition induced protection. Despite these interesting neuroprotective findings of SCD inhibition, the authors also report SCD inhibition toxicity in early (but not late) primary cultures, presumably because they are less reliant on MUFA synthesis and OLA. Results with human neuron cultures were similar to that of primary rat neuron cultures, including a differential response to SCD inhibition induced toxicity, associated with in vitro age, and that non-toxic concentrations do protect neurons from asyn stress.

The manuscript is very well-written and it includes thorough analysis. The abstract and title are representative of the findings. Appropriate statistics has been used and overall the presentation of data is good. Proper dose responses are appreciated for the inhibitor treatments which revealed how high doses of the SCD inhibitors are generally toxic.

These should be corrected before the study can be accepted for publicaiton:

1. Often overexpression alpha-synuclein causes formation of synuclein positive puncta that are soluble alpha-synuclein associated with vesicles/membranes, which is not an inclusion similar to what is seen in PD. Also, it is possible there is an increase in soluble pSyn in the aSyn 3K model, but not insoluble, beta-sheet pathologic alpha-synuclein. It would be helpful to show a better characterization of the “inclusions”. I suggest dot blots of homogenates with antibodies to synuclein monomer and beta-sheet oligomers (PMID: 32971232). Are the inclusions insoluble in Tx100? Cells could be fixed simultaneously with paraformaldehyde and 1% Tx100 and immunofluorescence performed for pSyn. Or, sequential extractions with Tx 100 followed by SDS and western blots would demonstrate shifts in solubility.

2. Since most PD subjects have synuclein pathology when they first see a neurologist, it would be helpful to know if the SCD inhibitors are protective after pathology is induced. It would be informative to see an experiment in which aSyn 3K expression is induced first (and wt aSyn as a control) followed by treatment with inhibitors.

3. Data were displayed as boxplot or line charts showing error bars with standard deviations (SD)’ --> It is suggested to use box-and-whisker plots that indicate all elements (e.g. median, interquartile interval, minimum, maximum) for all graphs.

4. Since most PD subjects have synuclein pathology when they first see a neurologist, it would be helpful to know if the SCD inhibitors are protective after pathology is induced. It would be nice to see an experiment in which aSyn 3K expression is induced first (and wt aSyn as a control) followed by treatment with inhibitors.. Fig. 2 a and c --> It is suggested to indicate between which groups the significance level relates to, like the authors do in Fig. 2 b and d.

5. Fig 2g: The difference between curve CAY and MF is not visible.

6. Fig. 4/5: Hoescht --> ‘Hoechst’

7. Fig. 4-6 and Extended data Fig. 2: the box plots could have a thinner outline to increase visibility of data representation.

8. It is advised to use the same type of box plot representation throughout the figures -->

Extended data Fig. 2 and 3: not all elements of the plot are visible when the box is ‘filled up’,

Extended data Fig. 5 is more clear in that regard.

9. Extended data Fig. 4 is missing?

10. Astrocytes in both the early and late primary cultures were more susceptible to aSyn 3K stress than neurons, this is clear in the early cultures where most death is in the astrocyte population (Fig 5A, B).’ --> Does this imply that this model is more related to MSA pathology than PD pathology? Has SCD gene alterations been linked to MSA in regard to protect against asyn pathology in astrocytes / oligodendrocytes?

11. In addition, the revised manuscript should be strictly in the format of eNeuro including the sections related to statistics and stastical table, RRIDs, eNeuro citing, see https://www.eneuro.org/content/preparing-manuscript#organization . An example how to make the statistical table can found for example in here, https://www.eneuro.org/content/8/1/ENEURO.0330-20.2020#T1
