# Supplementary figures and images for: SCD Inhibition Protects from α-Synuclein-Induced Neurotoxicity But Is Toxic to Early Neuron Cultures
Source: eNeuro. 2021 Aug 7;8(4):ENEURO.0166-21.2021. doi: 10.1523/ENEURO.0166-21.2021 (PMC8387157; doi:10.1523/ENEURO.0166-21.2021)

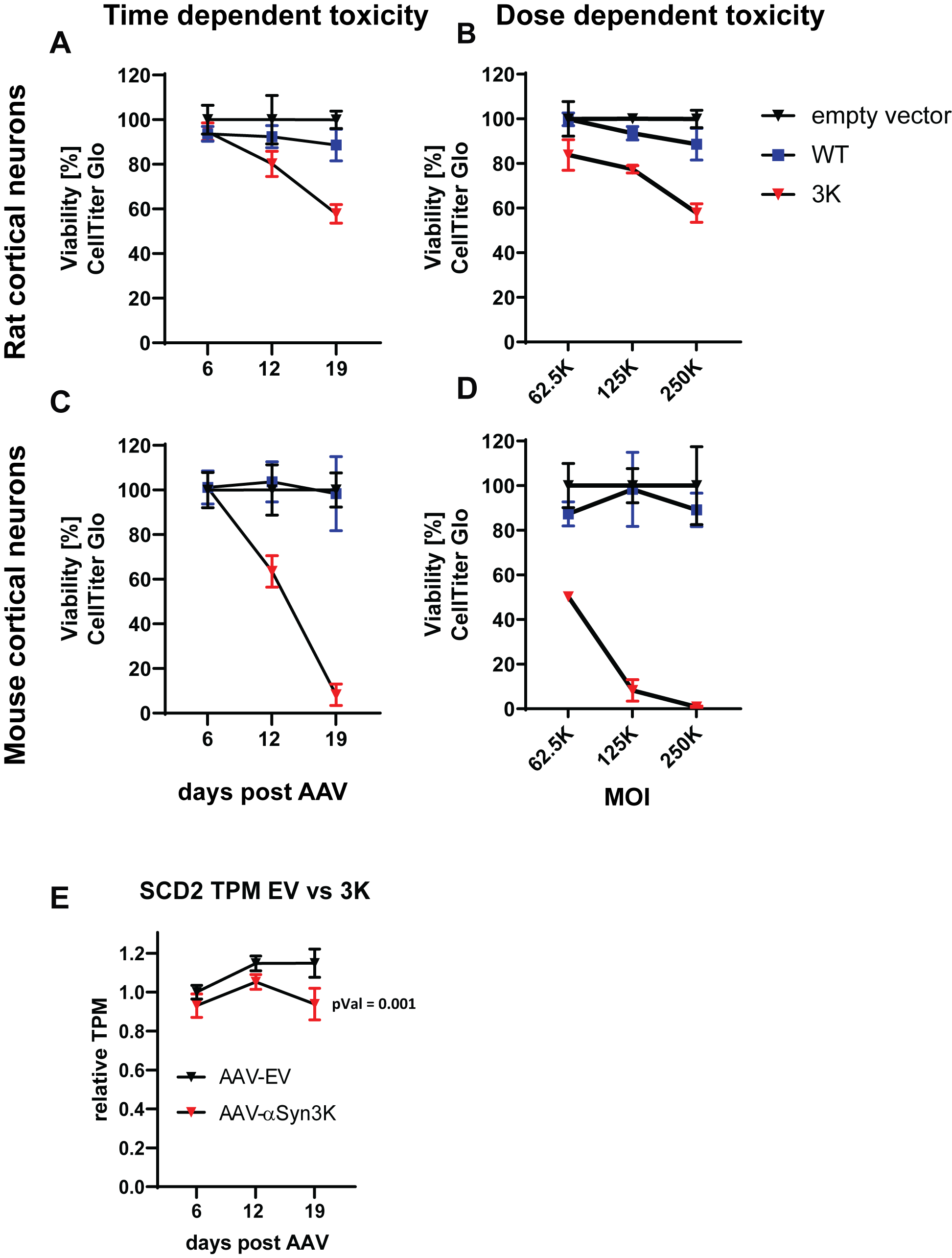

Supplement: Extended Data Figure 1-1 — αSyn WT versus 3K toxicity in rat and mouse cortical neuron cultures. Time (A) and dose (B; MOI of 62.5K, 125K, or 250K)-dependent toxicity measured by CellTiter-Glo in primary rat cortical neuron cultures treated with AAV9-EV or AAV9-αSyn 3K. C, D, Same as A, B except carried out in mouse cortical neuron cultures. Time-dependent plots A, C were done with AAV9 MOI of 250K. Dose-dependent plots B, D were assessed 19 d after AAV9 transduction. Note that AAV9-αSyn 3K transduction is always more toxic than WT or EV control. E, Relative transcripts per million of SCD2 transcripts in AAV9-EV and AAV9-αSyn 3K samples. Data displayed as line charts showing error bars with SD. Download Figure 1-1, TIF file. [file enu-eN-NWR-0166-21-s01.tif]

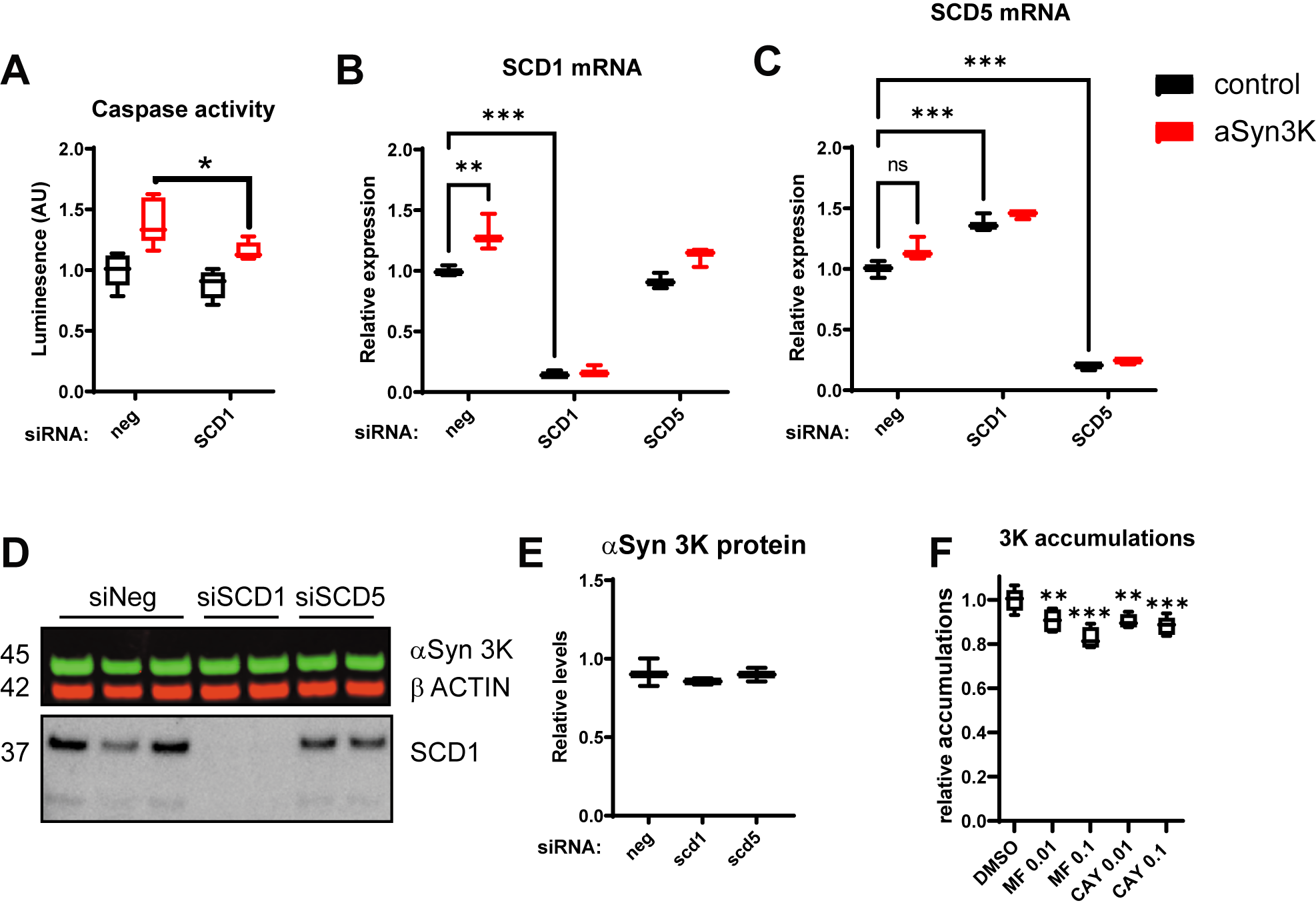

Supplement: Extended Data Figure 2-1 — siRNA knock-down of SCD characterization in αSyn 3K-GFP neuroblastoma model. A, Relative caspase activation under 5 nm siRNA knock-down of SCD1 in 3K neuroblastoma model. Note the rescue of αSyn 3K-GFP accumulation induced toxicity and caspase activation with SCD1 knock-down. B, Relative expression of SCD1 under 5 nm siRNA knock-down of SCD1 or SCD5. C, Same as B except SCD5 expression. Note that knock-down of SCD1 increases expression of SCD5. D, Representative Licor Western blotting depicting αSyn 3K-GFP, βactin, and SCD1 protein levels from samples treated as in A–C. siRNA treatment was performed 6 h before induction of αSyn 3K, all assessments were done 48 h after induction. E, Quantification of αSyn 3K-GFP protein levels as depicted in D, note that siRNA knock-down does not alter 3K protein levels. F, Accumulations were induced for 2 d before 1 d of SCD inhibitor (CAY or MF) treatment, n = 3 technical replicates. One-way or two-way ANOVA run with Dunnett’s multiple test correction, all data displayed as boxplots, *p < 0.05, **p < 0.01, ***p < 0.001. All plots n ≥ 3 independent experiments unless indicated otherwise. Download Figure 2-1, TIF file. [file enu-eN-NWR-0166-21-s02.tif]

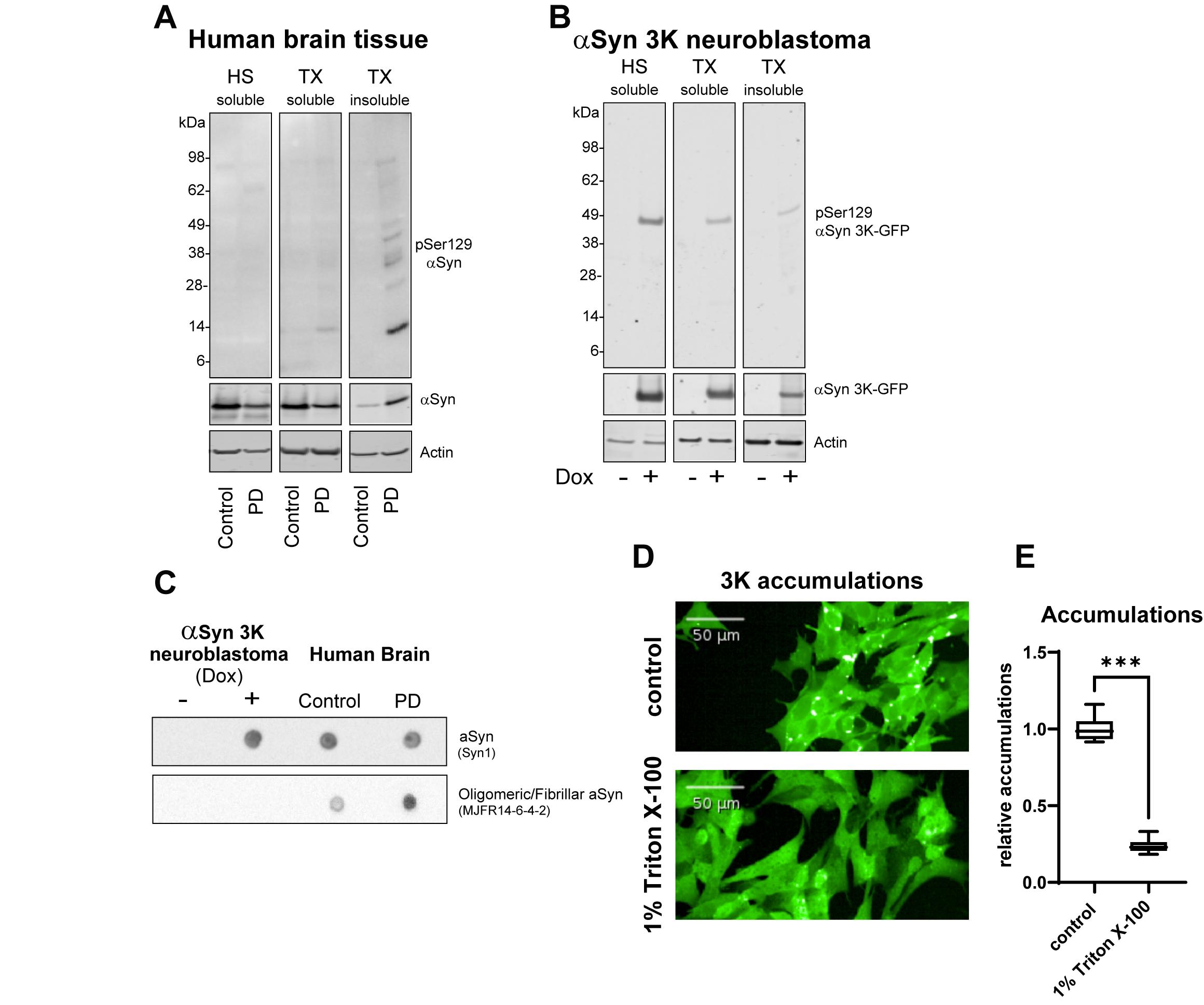

Supplement: Extended Data Figure 2-2 — Triton X-100 solubility of αSyn 3K-GFP accumulations. Sequential extraction with 750 mm NaCl (HS), 1% Triton TX-100 (TX soluble), and 1% SDS (TX insoluble) buffer in equal volume of (A) amygdala from control donor and donor with PD and (B) M17 cells overexpressing αSyn 3K GFP under Dox-inducible promoter. Western blotting for αSyn (MJFR1 at 1:1000 for A and 1:50,000 for B) and pSer129 αSyn (EP1536Y at 1:1000 for A and 1:25,000 for B). C, Dot blot analysis probing total extracts of M17 cells overexpressing αSyn 3K GFP (+/–Dox), control and PD extracts with total αSyn (Syn-1 1:1000) and oligomeric/fibrillar αSyn antibody (MJFR 14-6-4-2 1:10,000). D, Representative images of samples from E. E, Relative accumulation levels in 4% PFA fixed neuroblastoma cells with and without 1% Triton X-100 permeabilization, n = 18 technical replicates, Student’s t test; *p < 0.05, **p < 0.01, ***p < 0.001. Download Figure 2-2, TIF file. [file enu-eN-NWR-0166-21-s03.tif]

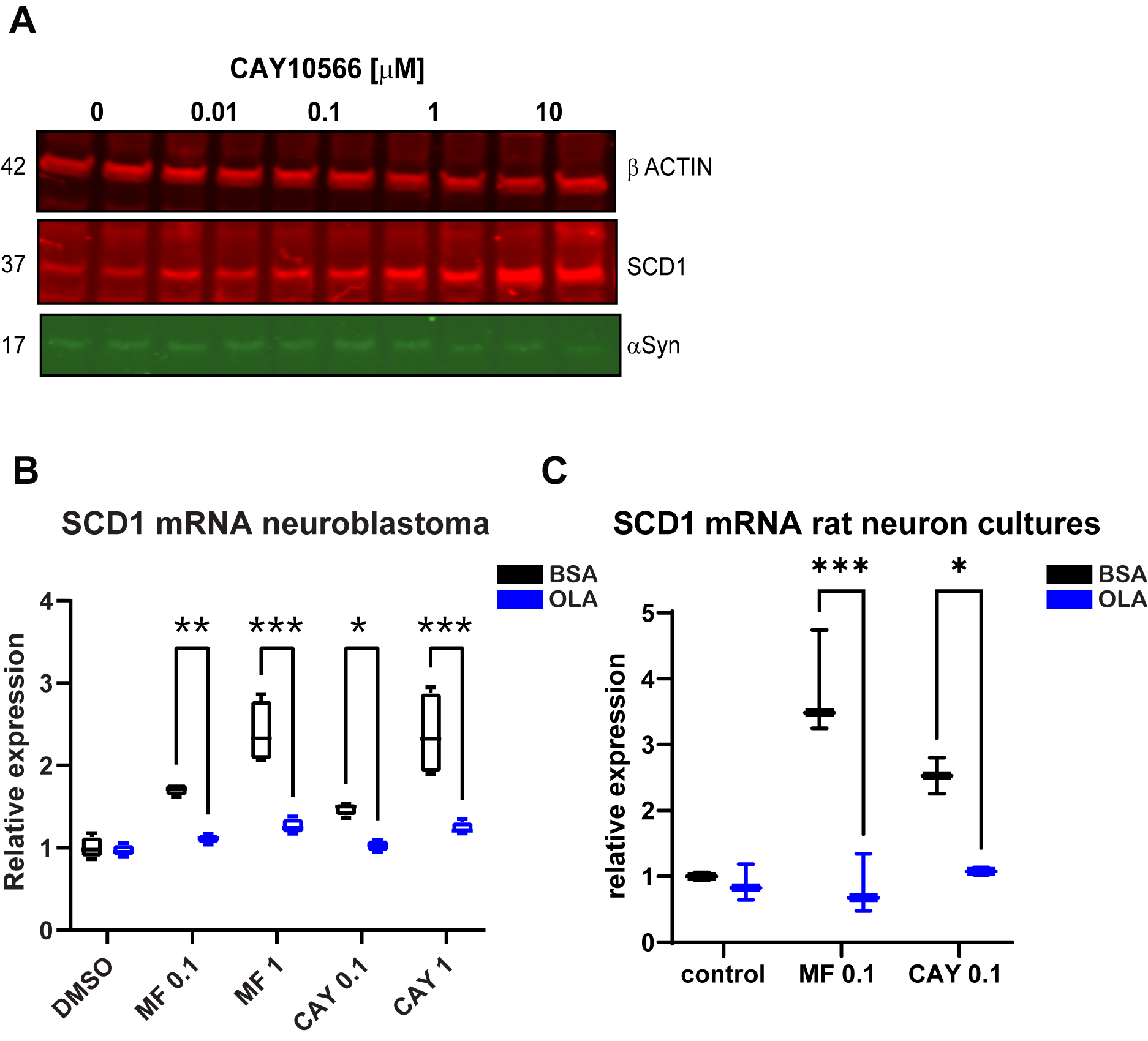

Supplement: Extended Data Figure 3-1 — SCD1 mRNA compensatory upregulation in neurons and neuroblastoma cells under SCD inhibition. A, Representative Licor Western blotting depicting endogenous SNCA (αSyn), βactin, and SCD1 protein levels from neuroblastoma cells, SCD inhibitor treatment was 48 h before protein isolation. B, Relative SCD1 mRNA levels in neuroblastoma αSyn 3K-GFP model under 0.01 or 0.1 μm SCD inhibition ± 100 μm OLA (48-h treatments). C, Relative SCD1 mRNA levels in primary rat cortical neuron cultures under 0.01 or 0.1 μm SCD inhibition ± 100 μm OLA (12-d treatments starting in DIV7 early cultures). Note that SCD1 mRNA increases under SCD inhibitor in both primary neurons and neuroblastoma cells and that this increase is reversed by OLA. Two-way ANOVA run with Dunnett’s multiple test correction, all data displayed as boxplots, *p < 0.05, **p < 0.01, ***p < 0.001. Download Figure 3-1, TIF file. [file enu-eN-NWR-0166-21-s04.tif]

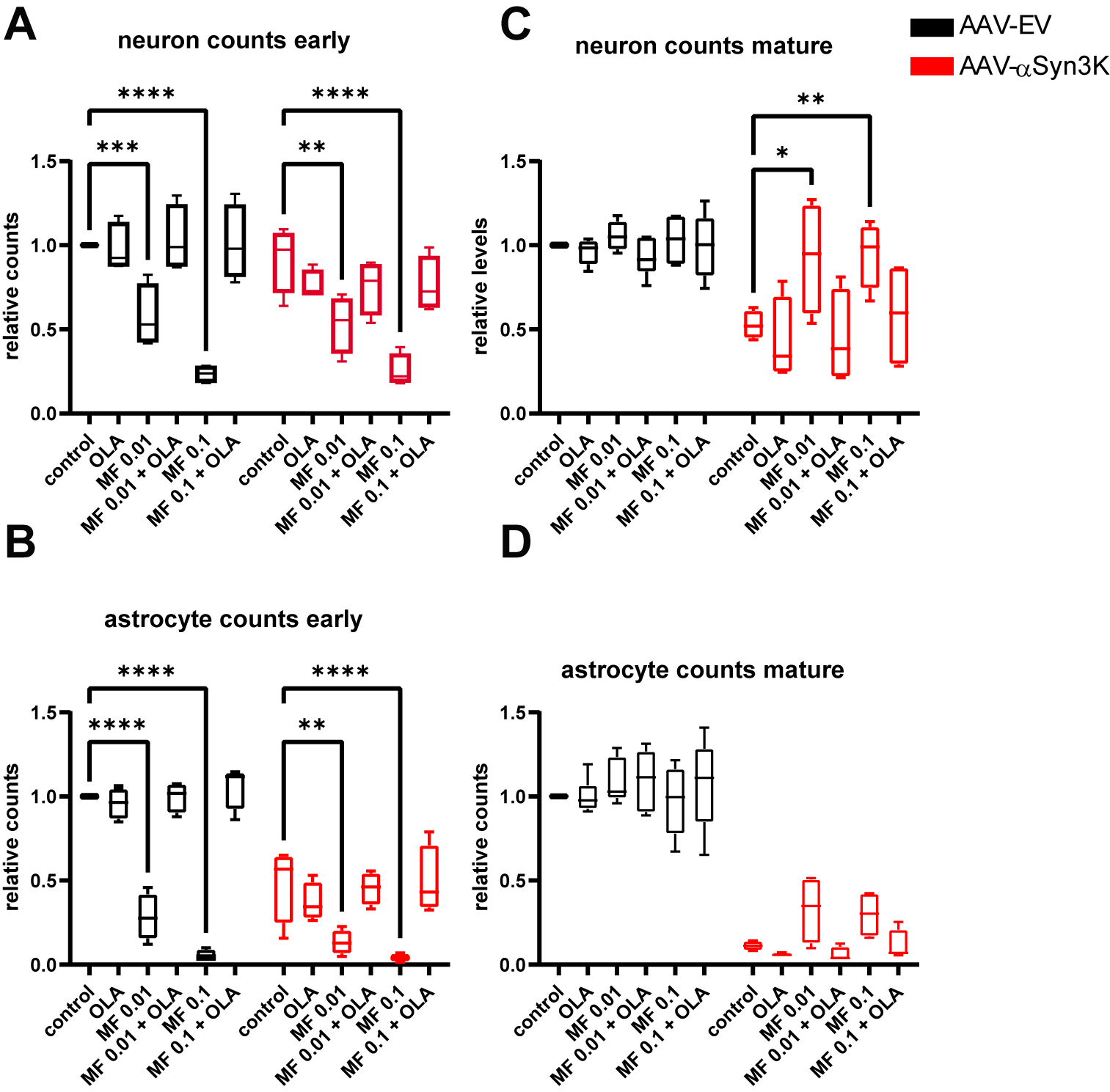

Supplement: Extended Data Figure 5-1 — Late primary neuron cultures are protected from αSyn 3K toxicity by SCD inhibition (MF). A, Neuron counts from early primary rat cortical neuron cultures treated with MF (0.01 or 0.1 μm) ± OLA (100 μm) + AAV9-EV or AAV9-αSyn 3K for 12 d. B, Same as A except treatments starting in late cultures. C, Astrocyte counts from early cultures. D, Astrocyte counts from late cultures. Experimental paradigm for A–D is equivalent to Figure 5, with the addition of AAVs and high-content image counting with MAP2 and GFAP markers for neurons and astrocytes, respectively. E, Representative high-content images of late cultures as in B, D. Note that late cultures are rescued from αSyn 3K toxicity by SCD inhibition, which can be reversed by OLA treatment. Two-way ANOVA run with Dunnett’s multiple test correction, all data displayed as boxplots, *p < 0.05, **p < 0.01, ***p < 0.001, ****p < 0.0001. All plots n ≥ 3 independent experiments. Download Figure 5-1, TIF file. [file enu-eN-NWR-0166-21-s05.tif]

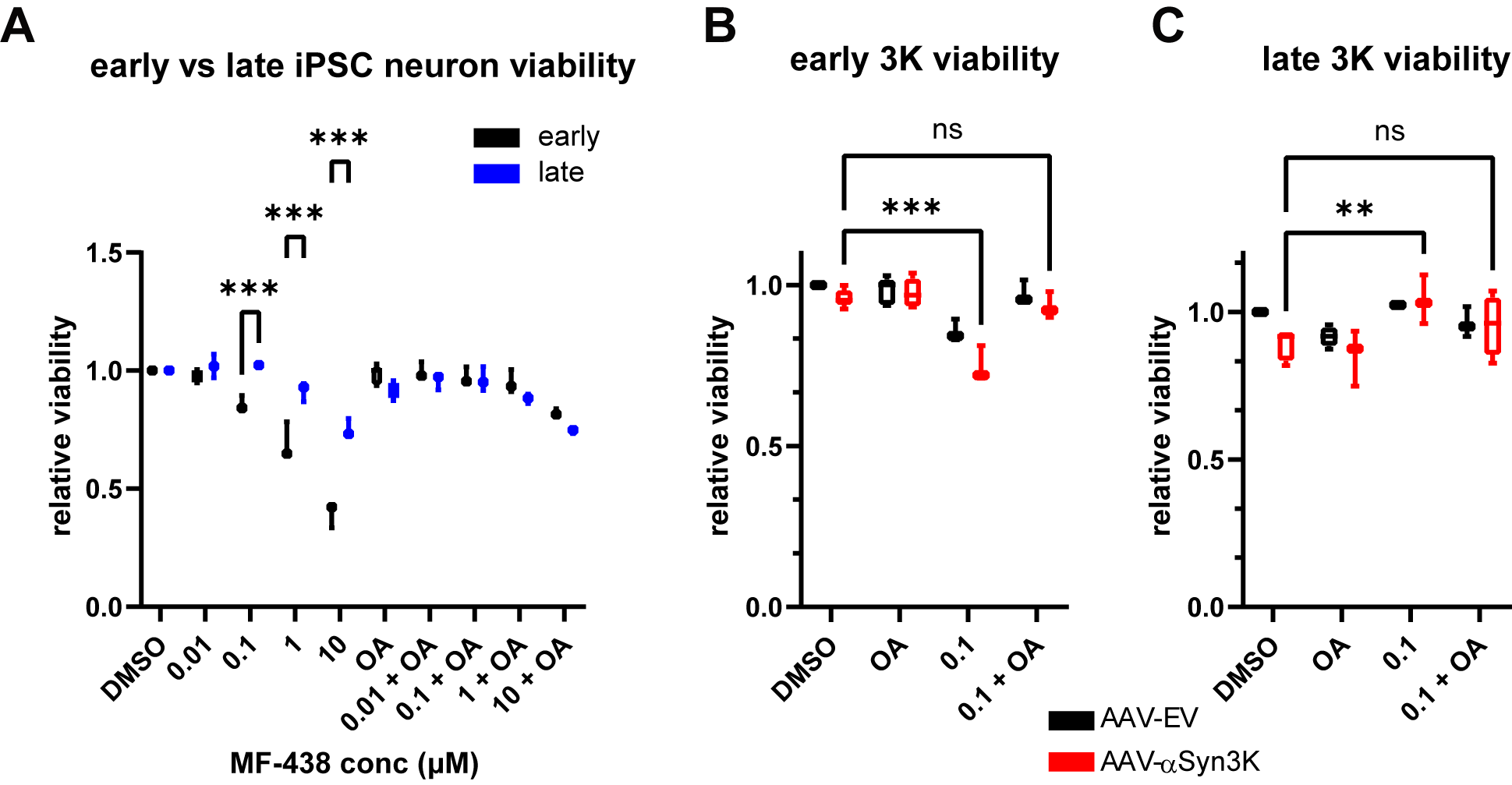

Supplement: Extended Data Figure 6-1 — Late human iPSC neurons are protected from αSyn 3K toxicity by SCD inhibition (MF) and are less sensitive to SCD inhibitor toxicity. A, CellTiter-Glo analysis on human iPSC neuron cultures after 12-d treatment ± MF (0.01, 0.1, 1, or 10 μm) ± OLA (10 μm) starting at DIV7 (early) or DIV21 (established). B, C, Same as A but only with 0.1 μm MF and with AAV9-EV or AAV9-αSyn 3K for 12 d in early (B) or late (C) cultures. Note the rescue of viability in late iPSC neuron cultures by 0.1 μm MF (a concentration non-toxic to establish neurons but toxic to early neurons). Two-way ANOVA run with Dunnett’s multiple test correction, all data displayed as boxplot, **p < 0.01, ***p < 0.001. All plots n ≥ 3 independent experiments. Download Figure 6-1, TIF file. [file enu-eN-NWR-0166-21-s06.tif]

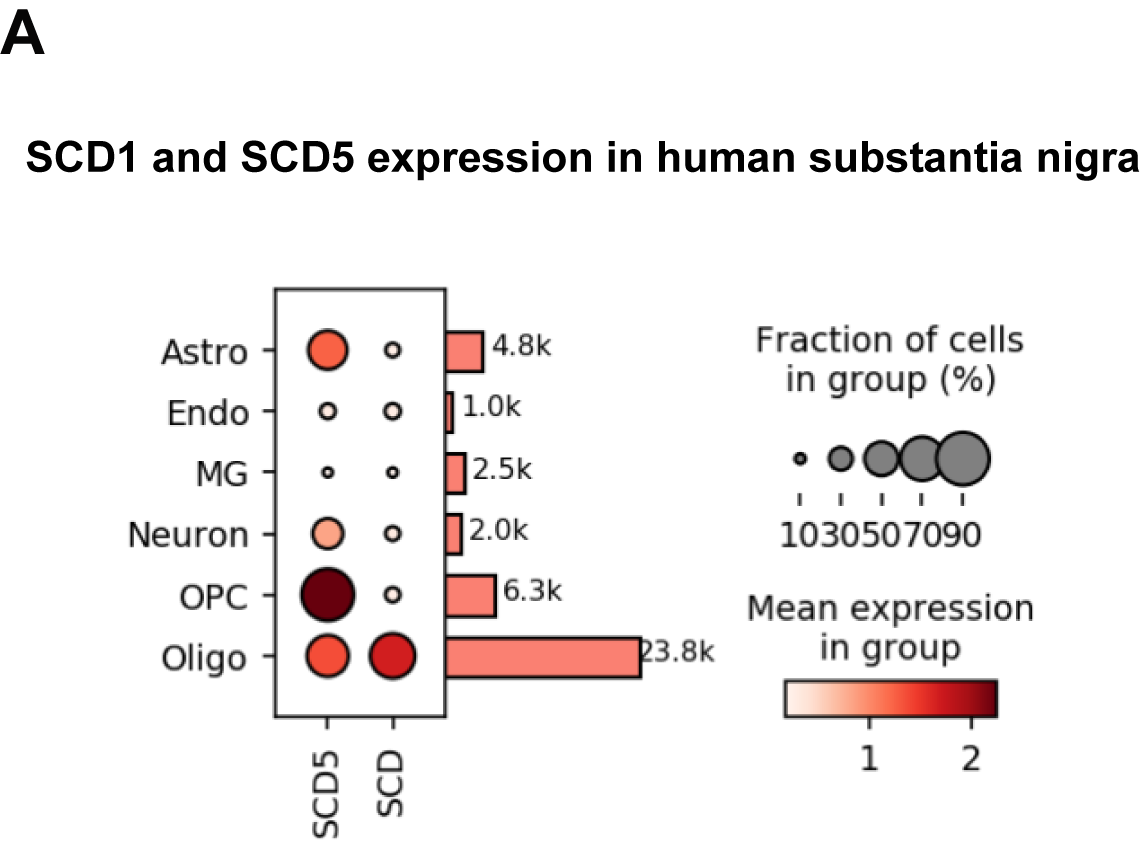

Supplement: Extended Data Figure 6-2 — SCD1 and SCD5 cell type-specific expression in human substantia nigra. A, Relative expression of SCD1 and SCD5 from human substantia nigra single-cell RNA-Seq (Agarwal et al., 2020). Note that the expression of the SCD5 isoform is higher than SCD1 in all but the oligodendrocyte population. Astro, astrocytes; Endo, endothelial cells; MG, microglia; Neuron, neuron; OPC, oligodendrocyte precursor cells; Oligo, oligodendrocyte. Download Figure 6-2, TIF file. [file enu-eN-NWR-0166-21-s07.tif]
